# E-Health Practices and Technologies: A Systematic Review from 2014 to 2019

**DOI:** 10.3390/healthcare9091192

**Published:** 2021-09-10

**Authors:** Maria Helena da Fonseca, Fanny Kovaleski, Claudia Tania Picinin, Bruno Pedroso, Priscila Rubbo

**Affiliations:** 1 Department of Production Engineering, Federal University of Technology—Paraná (UTFPR), Ponta Grossa 84017-220, Brazil; fannyk92@hotmail.com (F.K.); claudiapicinin@utfpr.edu.br (C.T.P.); 2 Division of Physical Education, State University of Ponta Grossa—Paraná (UEPG), Ponta Grossa 84030-900, Brazil; prof.brunopedroso@gmail.com; 3 Department of Accounting Sciences, Federal University of Technology—Paraná (UTFPR), Pato Branco 85503-390, Brazil; priscilarubbo@utfpr.edu.br

**Keywords:** e-health, ehealth, practices, technologies

## Abstract

E-health can be defined as a set of technologies applied with the help of the internet, in which healthcare services are provided to improve quality of life and facilitate healthcare delivery. As there is a lack of similar studies on the topic, this analysis uses a systematic literature review of articles published from 2014 to 2019 to identify the most common e-health practices used worldwide, as well as the main services provided, diseases treated, and the associated technologies that assist in e-health practices. Some of the key results were the identification of the four most common practices used (mhealth or mobile health; telehealth or telemedicine; technology; and others) and the most widely used technologies associated with e-health (IoT, cloud computing, Big Data, security, and systems).

## 1. Introduction

Digital change has been particularly challenging in healthcare, as there is growing demand for services due to the aging population and the emergence of new diseases. Thus, investment in new treatments is necessary so that everyone has equal access to the healthcare system [1,2,3]. E-health involves practices such as mhealth and telehealth that employ electronic technologies to provide healthcare resources, services, and information [4].

Mobile health or mhealth consists of the use of mobile devices so that patients can solicit services electronically, use apps to verify information, and manage or monitor treatment or problems or other health-related issues [5]. Telehealth can be defined as the use of telecommunication technologies to promote the care and education of patients and professionals working in the area [6].

E-health has become an integral part of the healthcare system as it addresses a range of difficulties in medicine, including reducing errors and providing more efficient services with more accurate results [7]. Such is the case with the use of electronic medical records, in which all information about a patient is stored, thus preventing inappropriate administration of medication during medical care and ensuring that the patient is treated quickly and comfortably [8]. However, its implementation depends on adequate planning and strategies so that virtual medical care can be performed [3].

The success of e-health in a country is related to several factors, including user acceptance and the types of infrastructure, systems, and management used [6,9]. Meanwhile, there are four stakeholders involved in the outcomes: entrepreneurs, healthcare professionals, patients, and those responsible for health insurance and assistance policies [10]. To effectively implement the use of information technologies in healthcare, e-health strategies must occur in an integrated manner, including the development of norms, laws, or regulations. This situation is valid whether in the fields of telehealth and mhealth, or specific categories such as electronic medical records or health literacy—eLearning (learning in health) [5,11,12].

The e-health strategy has three main components: (i) knowledge management; (ii) tools and methods; and (iii) policies. These components work to consolidate healthcare systems with support networks and scientific and technological production, manage infrastructure and human resources, reduce barriers to accessing health services, and promote community inclusion [13]. Some barriers to e-health include the difficulties faced when using systems and applications, both by healthcare professionals and patients, as well as ensuring the security and privacy of user data transmitted throughout these systems [12].

Another challenge in e-health is interoperability across systems. That is, new e-health systems must interact with existing ones, and there should be a standard electronic language between hospitals (or clinics) to facilitate communication and data exchange, as well as formal agreements on how the system should work in a standardized way [14,15]. Further, the cost of implementing e-health also presents challenges, which may make the implementation of such systems unfeasible. This is related to the high levels of investment required to purchase equipment to implement more sustainable practices [3,16] than traditional systems that store paper records, alongside the costs of hiring specialized support personnel in information technology (IT) to keep the systems running and software acquisition [8,17].

There are several studies on the topic of e-health, but they generally address a specific practice. One example is [18], in which the authors analyze studies of interventions via e-health using websites and social media in the treatment of patients with mood disorders. Two e-health-related studies with a general approach were found [19,20]. However, such studies do not fully address all e-health practices, which differentiates the present study from previous work on the topic. Although scientific publication databases have a vast range of studies on e-health [21], there are gaps in knowledge related to this topic, which justifies the need for the present review. Herein, we update data related to practices in the field of e-health and provide an overview of the information present in selected articles within a five-year time frame (2014 to 2019), demonstrating what has been published on the topic both in practical studies and literature reviews.

Therefore, the objective of this analysis is to identify, through a systematic literature review, the most commonly used e-health practices worldwide, as well as the key services provided, diseases treated, and the associated technologies that assist in providing e-health practices. This study aims to answer the following questions:

- In which countries and journals are studies published on e-health practices?
- What are the main e-health practices used worldwide?
- What are the main service delivery types and medical fields addressed using e-health?
- What are the main barriers to e-health service delivery?
- What are the most common diseases treated, and in which countries have e-health practices been applied?
- What are the most common technologies used in e-health?

## 2. Materials and Methods

The identification of the study portfolio was carried out using the Methodi Ordinatio process developed [22] and based on the Cochrane model and ProKnow-C for research studies that use Information and Communication Technologies—ICTs, such as spreadsheets, word processors, and reference managers. The following software programs were employed to conduct the analyses: Mendeley version 1.19.3; JabRef version 3.3; Microsoft Office 365 (Excel and Word); and NVivo trial version 11 for initial article analysis. The Methodi Ordinatio includes nine steps and is used to conduct systematic literature reviews, build bibliographic portfolios, and map the literature on a specific topic. One advantage of the methodology is its multi-criteria decision-making model that considers the impact factor of the journal, the number of citations, and the year of publication. From this, the InOrdinatio index is calculated, and the researcher obtains a bibliographic portfolio [23]. This methodology has been used by authors such as [24,25,26].

**Figure 1:**
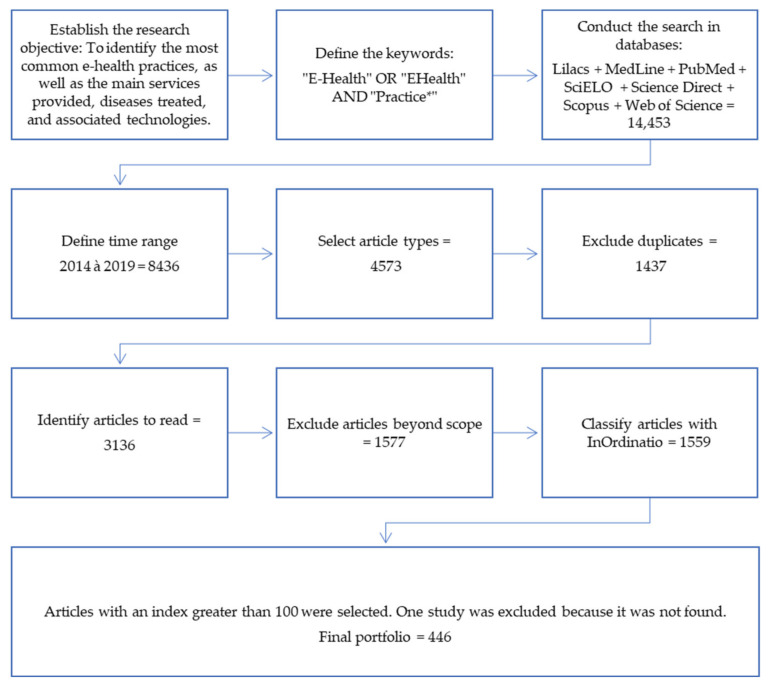
Research method.

The Methodi Ordinatio is shown in Figure 1 and is composed of the following steps:

1. Step 1—Establish the research objective;
2. Step 2—Define the keywords;
3. Step 3—Select the databases to be searched;
4. Step 4—Search for and register the results obtained;
5. Step 5—Define search filters such as publication period and type of study (articles, books, etc.), excluding duplicate articles and those unrelated to the selected research topic;
6. Step 6—Identify the impact factor, year of publication, and number of citations of the articles from Google Scholar for the selected portfolio;
7. Step 7—Apply the InOrdinatio equation [22] in a spreadsheet to classify the identified articles;
  (1) $$\mathrm{InOrdinatio}=\left(IF÷1000\right)+\alpha \times \left[10-\left(\mathrm{ResearchYear}-\mathrm{PublishYear}\right)\right]+(\sum Ci)$$
  The equation is composed of the following variables: *IF* is the impact factor; *α* is the value defined by the researchers considering the current relevance of the articles, which may vary from 1 to 10 (for this study, we defined *α* as 10); ResearchYear is the year the research was conducted; PublishYear is the year the article was published; and ∑*Ci* is the number of citations of the article according to data from Google Scholar.
8. Step 8—Select the articles for the final portfolio considering the highest InOrdinatio classification; and
9. Step 9—Read and analyze the final portfolio articles.

First, the search objective was defined, and the keywords were searched using Boolean operators (“E-Health” OR “EHealth” AND “Practice *”). The keyword “Technology” was not used to search the databases so as not to excessively limit the search. The analysis of e-health-related technologies took place after the portfolio was defined.

The search was carried out using seven electronic databases: Lilacs, MedLine, PubMed, SciELO, Scopus, Science Direct, and Web of Science. These databases were chosen due to the multidisciplinary character of this study and the fact that the information concerning medicine and health is vast. Thus, to conduct a careful analysis, well-known and key sources in the field should be used, such as MedLine, PubMed, Lilacs, SciELO, the Cochrane Library, and the Virtual Health Library (VHL).

MedLine is a database containing supplementary materials and offers a good basis for research as it indexes other databases and aggregates complementary materials. It has been employed in research on e-health, health, and medical technology [6].

Scopus is the largest research database for the fields of technology, medicine, science, social sciences, and the arts and humanities, while Science Direct contains technical publications in the fields of science and health [27]. The Web of Science is an independent global citation database that offers reliable multidisciplinary data for academic research [28].

The time frame for the published research was five years, from 2014 to 2019. According to [29], a five-year period is the half-life for article citations. In addition, the following filtering procedures were applied to the databases to obtain the results: selection of types of articles published (review, research, and journal-published), exclusion of duplicate articles. Thus, 3136 articles were obtained, whose titles, abstracts and keywords were read to exclude studies that dealt with areas unrelated to e-health practices and are beyond the scope of this analysis.

Finally, the Methodi Ordinatio equation was applied to obtain the most currently relevant articles. Articles with an index greater than 100 were considered, and one study was excluded as it was not found, resulting in a final portfolio of 446 articles, as listed in Appendix A—Table A1. Final portfolio.

The database search did not exclude any publication language, aiming to obtain a broad range of results and not limit the present analyses. Table 1 shows how the studies were analyzed to answer the research questions.

The questions referring to countries that are publishing studies on e-health and countries that use these practices were answered, respectively, by identifying the country of the first author and where the studies took place. Thus, the results and discussions of the research were organized in a bibliometric and content analysis carried out on the 446 articles from the final portfolio. For the content analysis of the technologies associated with e-health, 57 articles from the final portfolio (446 articles) were analyzed as they specifically address this topic.

## 3. Results and Discussion

### 3.1. Bibliometric Analysis

**Figure 2:**
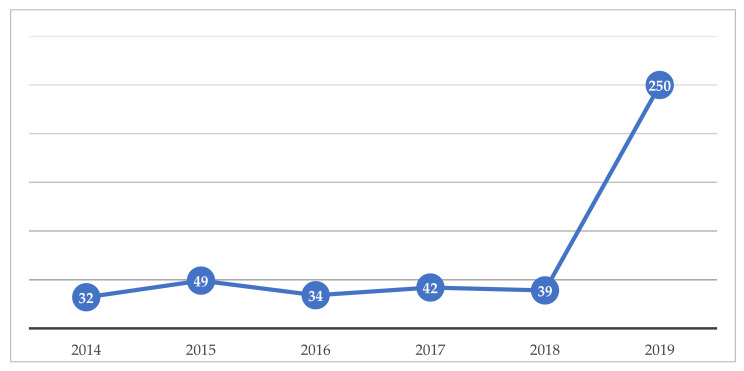
Number of publications per year.

Figure 2 shows the number of publications per year from 2014 to 2019.

We can see that 2014 had the lowest number of publications on the topic and 2019 the highest. There was fluctuation in the number of publications, particularly between 2018 and 2019, when a marked increase in the number of studies related to the theme of e-health occurred. This increase is related to several factors. The area of mhealth has been growing, as has the number of chronic diseases and other health problems, while the advance of mobile technology has enabled greater access to e-health through devices, enabling an increase in research [30]. An increase in publication on the topic is also due to researcher interest, as it is current and still novel, allowing for greater numbers of studies on topics within the field, as is the case with reports published about e-health indicators, such as those from the Global Health Observatory of the World Health Organization (WHO) and the Nordic e-Health Research Network [31].

**Figure 3:**
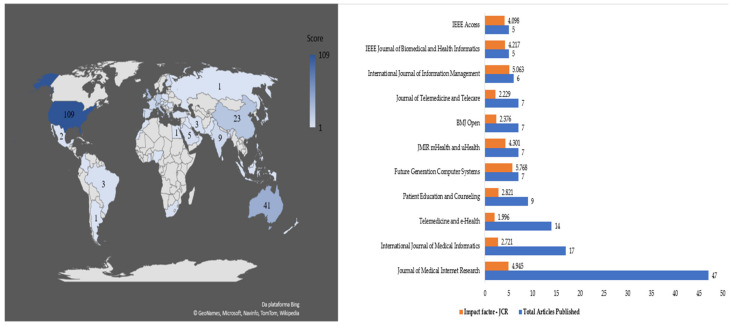
Distribution of articles by country and number of articles published by journal.

Although authors from 58 countries contributed to studies, the geographical distribution was uneven, as shown in Figure 3.

The leading countries in developing research related to e-health are United States (109), Australia (41), United Kingdom (32), China (23), Italy (19), Germany (18), Norway (14), and France (10). The countries with the fewest publication include Egypt (1), Mexico (2), and Brazil (3). In the study by [32] on e-health and health informatics skills, the authors found that the countries with the most authors who published on the topic were United States, Canada, United Kingdom, and Australia, and those with the least were Denmark and Norway.

Norway, Australia, Germany, the United Kingdom, United States, France, and Italy are countries with a remarkably high Human Development Index—HDI (which is based on the health, life expectancy, education, and income of a country’s population), while Egypt, Brazil, Mexico, and China are countries with high HDI. The wealthier a country is, the better its economic, political, and healthcare infrastructure, enabling more initiatives for e-health development, as has occurred in many European countries [33,34]. Each country has a different level of e-health development, and consequently, countries with more advanced e-health systems have a higher incidence of scientific production on the topic, such as European countries and the United States, as verified by [35]. Moreover, [36] state that although countries on the African continent and Latin America are publishing on the topic, the number of studies is still low as the countries have less developed e-health systems. Such is the case with Brazil and Mexico, which are still at the stage of telehealth improvement and consolidation.

The 446 articles in the portfolio were published across 253 academic journals. However, some journals among those identified stand out for having very few publications on the topic but a high impact factor, such as *The Lancet* (1 publication—60.392 JCR); *British Medical Journal*—BMJ (1 publication—30.223 JCR); *IEEE Communications Surveys & Tutorials* (1 publication—23.700 JCR); *IEEE Internet of Things Journal* (2 publications—9.936 JCR); *Schizophrenia Bulletin* (2 publications—7.958 JCR); *Information Sciences* (2 publications—5.910 JCR); *AIDS and Behavior* (3 publications—3.147 JCR); *Social Science & Medicine* (3 publications—3.616 JCR); *International Journal of Environmental Research and Public Health* (3 publications—2.849 JCR); *Journal of the American Medical Informatics Association*—JAMIA (4 publications—4.112 JCR); *Frontiers in Psychiatry* (4 publications—2.849 JCR); *PLoS One* (4 publications—2.740 JCR).

In the analysis by [19], who conducted a literature review on e-health, and [32], who did a bibliometric study, the journals that had the most publications were *Journal of Medical Internet Research* and *Telemedicine and e-Health*.

Therefore, for the research question “In which country and journals are studies on e-health practices published worldwide?” we can see that the United States stands out as it has the greatest number of authors who published on this topic, with 109 studies. Moreover, the *Journal of Medical Internet Research* is the most prominent scientific outlet for research on e-health, with 47 publications.

### 3.2. Content Analysis

#### 3.2.1. Main E-Health Service Delivery Types and Fields

Based on the survey of studies from the final portfolio, Table 2 presents the categories of practices and services provided in e-health as described in the analyzed studies.

When answering the research question “What are the most common e-health practices used around the world?” four categories of e-health practices became evident: (i) mhealth or mobile health; (ii) telehealth or telemedicine; (iii) technology; and (iv) others, which include combinations of different practices.

E-health can be defined as healthcare services and health information provided and/or obtained using the internet, mobile devices, computers, and information technology [64]. It involves the application of digital solutions for healthcare, thus facilitating patient care in a more comfortable way [62]. The practice areas are described below.

Mhealth or mobile health enables persons who use mobile devices, such as smartphones, to access systems, data, and apps to monitor and manage their health status [38]. Telehealth or telemedicine includes remote consultations via videoconference/call using desktop computers, smartphones, or tablets with internet access [44,67,68]. 

The technology applied in e-health helps healthcare professionals, patients, and the lay population to obtain information or access learning, treatment, and resources that are available online [69].

Through e-health, digital health interventions (DHIs), such as assisted therapy, have been shown as effective among children and young people undergoing mental health treatment, as discussed in the systematic review by [64] on randomized clinical trials of DHIs performed with children and young people up to 25 years of age. However, further data are required to offer satisfactory conclusions about the benefits; despite the potential that the intervention can offer, few studies have been carried out on the subject and with small sample sizes, making this assessment difficult.

In an analysis by [62], the authors evaluated the effectiveness of an electronic health project model for people with severe mental illness (SMI), called the Flat Explicit Design Model (FEDM). The study was carried out via a website with 38 people aged 31 to 59 years who have some degree of severe mental illness and involved online tests.

We also identified a study that sought to conduct a theoretical review on the services and applications offered in the practice of mhealth and their use for therapy in the areas of mental health and behavioral disorders, musculoskeletal and connective tissue systems, oncology, and the nervous system [38]. Another study assessed the skills of medical residents, doctors, and clinics working with telepsychiatry, with a focus on e-mental health (e-MH) care [44].

In the “other” category, articles describing a combination of past practices and other areas, such as costs, were included. The study by [56] assessed the current literature to verify whether specialization in telehealth offset the effective cost. The authors found specializations such as teledermatology, teleophthalmology, and telecardiology offset the amount invested in that healthcare service.

Some of the service delivery types and other themes combined with the most representative e-health practices in the literature are discussed below.

Health literacy is knowledge and skills related to health, and it can be obtained through in-person and/or online environments [70]. The latter includes websites, social media, and the use of health data systems [71]. An aspect related to this area in the analyzed studies is low health literacy, as seen in the studies by [72,73]; when searching for health information, internet users must use a critical approach to determine if the information obtained is correct. Often, the information posted online is presented erroneously, which may misguide or misinform the user about the content.

Mhealth or mobile health and monitoring consists of using smartphone applications to help in disease treatment, identification, and support [74]. It also encompasses health exam scheduling and patient monitoring performed via devices with wireless sensors that check essential vital signs, such as blood glucose level and blood pressure [38]. The results of these exams are stored on the device, thus allowing for the transmission of data from exams done at home [75].

In telehealth, when an intervention is combined with interaction with a healthcare professional, it can be defined as telemonitoring of patients with diseases that need follow-up [43]. This enables doctors to see patients without having to go to the hospital or doctor’s office [45]. Such a service delivery can be employed with mental health patients who require therapy [44], enabling them to participate in sessions at home at more flexible times [76]. It can also be used to monitor the symptoms of cancer patients undergoing treatment [77].

The systems related to the category “technology” entail the creation of encryptions to protect the data of patients who access their medical records online, as well as data protection in the systems of hospitals, pharmacies, and clinics [52,53] so that data remain confidential and secure [54]. Technology also involves creating support for the systems [78] and developing mobile devices that can be used in the healthcare sector, such as wearable sensors [79]. Further, it includes the use of Internet of Things (IoT) [80], cloud storage [81], and Big Data [82] in the areas of e-health.

Regarding the research question “What are the main service delivery types and medical fields served in e-health?” we found that the main areas of service provision are health literacy, mhealth and monitoring, telehealth, and systems combined with technology. In turn, the main medical fields served that are highlighted in the literature are telepsychiatry, teledermatology, telerehabilitation, teleophthalmology, telecardiology, and teledentistry.

As for the research question “What are the main barriers to e-health service delivery?” we found that costs, laws, and system data security of are the main barriers to be overcome. We identified studies related to e-health practices experiencing barriers in terms of costs related to the use of telemedicine [83], regulation and legislation [38], security in the use of cloud computing [84], limitations in clinical trials of existing digital interventions [64], and limitations in databases [58]. In mHealth, a limiting factor is mobile device battery life [85], as the devices with applications or monitoring equipment must have the battery charged to avoid any problems during use [86]. Further, geographic distance is a primary limiting factor, as each country has different health-related applications available to the population [87] and barriers between patient and physician [38].

#### 3.2.2. Main Diseases Treated

**Figure 4:**
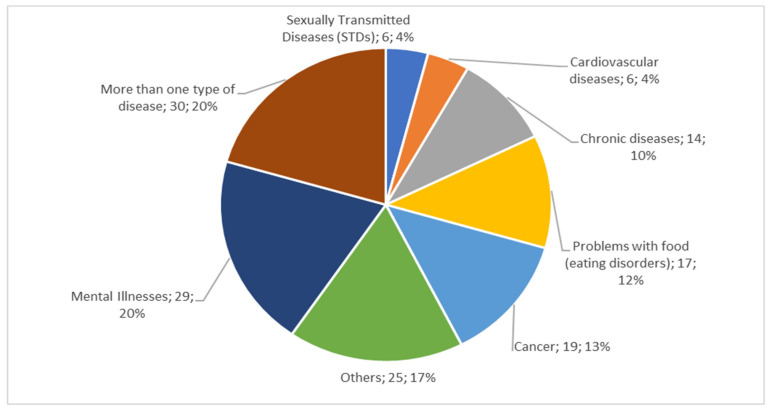
Diseases treated with e-health.

Figure 4 shows the main diseases treated in e-health (total and percentage), addressing the research question “What are the most commonly treated diseases and in which countries have e-health services been used?” The most commonly treated diseases are mental illnesses, multiple diseases (diabetes mellitus 1 and 2, stress, depression, and anxiety), cancer, eating disorders, chronic illness, cardiovascular diseases, and sexually transmitted diseases (STDs). Regarding the countries that currently use e-health, we identified the following countries where studies have been conducted: United States, Canada, Australia, Germany, Sub-Saharan Africa, Africa, Netherlands, Sweden, Switzerland, China, Italy, Greece, Finland, Iran, Iraq, Bangladesh, Pakistan, Saudi Arabia, United Kingdom, Spain, France, Italy, and Portugal.

Some of the studies evaluate more than one type of issue, for instance, diabetes mellitus (DM), cardiovascular diseases, and chronic lung diseases [39]; mental illnesses such as depression, anxiety, dementia [76]; others, such as the oral health of pregnant women in prenatal care, anti-smoking, organ transplants [88]; cancer, such as breast cancer, skin cancer, and lung cancer [77]; eating disorders, including care regarding infant nutrition, diabetes, and obesity in adults [75]; chronic illness, such as chronic inflammatory rheumatic diseases (CIRDs), asthma, and chronic pain [10]; cardiovascular diseases [89]; cerebrovascular accident (CVA), coronary artery disease, and atrial fibrillation (AF) [90]; sexually transmitted diseases (STDs), such as HIV and/or sexually transmitted infections (STIs) [50]; human papillomavirus (HPV) and child sexual abuse [91].

Mental illnesses and the studies combining more than one type of disease represented 40% of diseases treated with e-health addressed in the literature. The studies deal mainly with randomized clinical trials applied to groups of people. For example, in the study conducted by [92], the authors verified how a telerehabilitation program works to help control type 2 DM. In the study by [93], a randomized clinical trial (RCT) was carried out with 13 primary brain tumor patients. Using the ReMind App, the pilot study obtained favorable results from patients but still required improvement. On the other hand, [94] observed 66 stroke (CVA) patients and their caregivers for eight weeks, using an e-health system as a treatment aid to do physical exercise. They found no significant effect on patient improvement during that period.

Other authors have chosen to review randomized studies, such as the study by [95], who compared the estimated sizes of combined effects through meta-analyses of analysis of covariance (ANCOVA), simple analysis of change score (SACS), and simple analysis of final values (SAFV), using RCTs of digital interventions with glycated hemoglobin HbA1c as the main result or intervention for disease treatment using e-health practices. Finally, in the study by [96], the use of mhealth for intervention as a form of treatment for patients with hallucinations was investigated.

#### 3.2.3. Technologies Associated with E-Health

**Figure 5:**
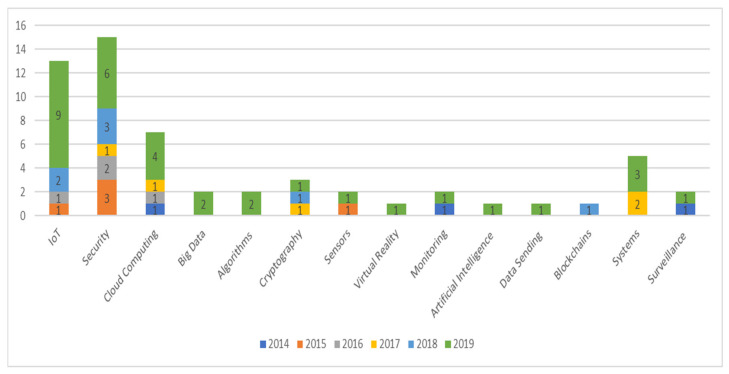
Number of publications in articles combining technology and e-health.

From the final portfolio (446 articles), 57 publications were selected that address topics related to technology applied in healthcare. When answering the question “What are the most used technologies in e-health?” we identified IoT, cloud computing, Big Data, security, cryptography, algorithms, among others, as shown in Figure 5.

The technology applied to healthcare includes IoT to integrate medical sensors [80], authenticate, encrypt [97], and maintain security and confidentiality during data exchanges [98] and between physicians and patients [99]. It is vital that users are confident that the systems used are protected [100] and authenticated to ensure the privacy of stored data [51,101]. The cloud system facilitates data storage at a low cost while also being secure and private [42,81,84].

The use of Big Data entails the analysis, diagnosis, and treatment of diseases [82,102]. There is also the use of wireless sensors applied to clothing, which can be used to monitor a patient when performing physical exercise [103,104,105].

The first authors of each of the 57 articles addressing technology in e-health are from 35 different countries, principally China (with 10 articles); India (6 articles); the United States, Australia, and Italy (3 articles each); and Greece, Algeria, Pakistan, and Saudi Arabia (2 articles each). Other countries, such as Spain, South Africa, and Mexico, had only one study. Articles related to technology were published in 38 journals, mainly *Future Generation Computer Systems* (7 articles); *IEEE Access* (4); *International Journal of Information Management* (4); *Journal of Medical Systems* (3); *Computers & Electrical Engineering* (2); *Health and Technology* (2); *Information Sciences* (1); and *Telematics and Informatics* (1). In the literature review by [106] on the application of cloud computing in e-health, 44 studies published between 2010 and 2013 were analyzed. The authors were from countries including the United States (11), Australia (4), China (3), India (2), and Spain (1). As we can see, the results found by [106] are similar to those reported herein.

Through an analysis of the literature, we found that technology can provide benefits across healthcare practices, such as the development of products, processes, and systems, as well as support decision-making. Nonetheless, data security and privacy remain a concern, both for developers and users; regardless of how efficient technologies are becoming, there can always be failures. Thus, there is room for in-depth studies and the development of technologies with more secure systems.

## 4. Conclusions

To achieve its proposed objective, the present study used a systematic literature review of articles published from 2014 to 2019, resulting in the analysis of 446 articles. Six research questions were defined to analyze the literature on e-health practices. Through the analysis, four categories of the most common practices in the field were identified: “mhealth or mobile health”; “telehealth or telemedicine”; “technology”; and “others.” The main services provided with e-health practices are health literacy, mhealth and monitoring, telehealth, and systems combined with technology. Regarding the fields of medical specialties that offer consultations via telehealth, the following were identified: telepsychiatry, teledermatology, telerehabilitation, teleophthalmology, telecardiology, and teledentistry. Other fields were also found that offer services in e-health, such as geriatrics, general medicine, endocrinology, pediatrics, gynecology, and oncology.

Furthermore, 146 of the 446 studies analyzed use one of the common practices for disease diagnosis/treatment/monitoring, with mental illnesses and studies combining the treatment of multiple disease corresponding to 40%. Among the most frequently treated diseases are diabetes mellitus, cardiovascular diseases, chronic lung diseases, and mental illnesses (depression, anxiety, dementia). In terms of the countries that current use e-health, countries in Europe (Austria and Germany), Africa (Sub-Saharan countries), Asia (China), and North America (United States and Canada) stand out.

In relation to the technologies adopted in e-health practices, IoT, cloud computing, Big Data, security, and systems are among the most common.

Systemic reviews such as the one presented herein are important to provide more precise targeting of the practices and technologies that can be adopted in different procedures and processes, considering the context of each country. In general, such analyses can offer information and guide physicians and patients in the use of “new” services.

A study can always be improved, and the present one is no exception. Although the methodology applied followed careful steps and well-defined filters, the choice of only three keywords (considered comprehensive) may have resulted in the exclusion of some relevant studies in the analysis.

The present study was completed at the beginning of the global COVID-19 pandemic, during which physicians and patients began to use considerably more e-health practices and services due to the restrictions imposed by social isolation. Further studies are needed to address the growth in e-health practices, including services, diseases, and new approaches that are in greater demand, and the breakdown of government-imposed restrictions on expanding the use of e-health.

## Figures and Tables

**Table 1:** Type of analysis used to address the research questions.

Type of Analysis	Research Question
Bibliometric analysis	In which countries and journals are studies on e-health practices published?
Content analysis	What are the main e-health practices used worldwide? What are the main service delivery types and medical fields served with e-health? What are the main barriers to e-health service delivery? What are the most commonly treated diseases, and in which countries has e-health been applied? What are the most common technologies in e-health?

**Table 2:** E-health practices and fields.

E-Health Practices	Specification of Service Delivery Types and Other E-Health-Related Topics Found in the Articles	Medical Fields Identified in the Articles	Authors
Mhealth or Mobile Health	Mhealth; assessment; systems assessment; information; telehealth; monitoring; health literacy.	General medicine; emergency; pediatrics; cardiology; oncology; psychiatry; neurology; dermatology; gynecology; hematology; infectiology/infectious diseases; radiotherapy/radiology; diagnostic imaging; gastroenterology; anesthesiology; nutrition; orthopedics; respiratory system/otorhinolaryngology; general surgery; urology; geriatrics; endocrinology; nephrology; ophthalmology.	[37,38,39,40,41,42]
Telehealth or Telemedicine	Telehealth; intervention; interaction; mhealth; systems; technology.	Telepsychiatry; teledermatology; teledentistry; telerehabilitation; teleophthalmology; telecardiology.	[43,44,45,46,47,48]
Technology	Technology; others; mhealth; patient monitoring; support; systems; telehealth; knowledge level; health literacy; systems assessment; program.		[49,50,51,52,53,54]
Others	Others; diagnosis; telehealth; mhealth; costs; programs; problems; project; quality of services; general e-health; care; project assessment; benefits; development; diagnosis; evaluation system; impact x cost-benefit; use and acceptance/barriers; knowledge level; mhealth; treatments; professionals’ views; study groups; intervention assessment; health literacy; technology; telehealth; randomized controlled trial—RCT; standardized service.	Nursing; oncology; gynecology; psychology; neurology; cardiology; psychiatry.	[55,56,57,58,59,60,61,62,63,64,65,66]

## Appendix A

**Table A1:** Final portfolio.

Author	Title	Journal	Impact Factor	Year	Citations	InOrdinatio 10
Stoyanov, S.R.; Hides, L.; Kavanagh, D.J.; Zelenko, O.; Tjondronegoro, D.; Mani, M.	Mobile app rating scale: A new tool for assessing the quality of health mobile apps	*JMIR mHealth and uHealth*	4.301	2015	550	550.00
[38] Silva, B.M.C.; Rodrigues, J.J.P.C.; Torre-Díez, I.D.L.; Lopez-Coronado, M.; Saleem, K.	Mobile-health: A review of current state in 2015	*J. Biomed. Inf.*	2.950	2015	454	514.00
[83] Flodgren, G.; Rachas, A.; Farmer, A.J.; Inzitari, M.; Shepperd, S.	Interactive telemedicine: Effects on professional practice and health care outcomes	*Cochrane Database Syst. Rev.*	7.755	2015	245	305.01
[39] Whitehead, L.; Seaton, P.	The effectiveness of self-management mobile phone and tablet apps in long-term condition management: A systematic review	*J. Med. Internet. Res.*	4.945	2016	206	276.00
Sultan, N.	Making use of cloud computing for healthcare provision: Opportunities and challenges	*International Journal of Information Management*	5.063	2014	202	252.01
[105] Wang, J.B.; Cadmus-Bertram, L.A.; Natarajan, L.; White, M.M.; Madanat, H.; Nichols, J.F.; Ayala, G.X.; Pierce, J.P.	Wearable sensor/device (Fitbit One) and SMS text-messaging prompts to increase physical activity in overweight and obese adults: A randomized controlled trial	*Telemed. e-Health*	1.996	2015	173	233.00
Fortino, G.; Galzarano, S.; Gravina, R.; Li, W.	A framework for collaborative computing and multi-sensor data fusion in body sensor networks	*Information Fusion*	10.716	2015	167	227.01
Boudreaux, E.; Waring, M.; Hayes, R.; Sadasivam, R.; Mullen, S.; Pagoto, S.	Evaluating and selecting mobile health apps: strategies for healthcare providers and healthcare organizations	*Transl. Behav. Med.*	2.237	2014	173	223.00
[3] Ross, J.; Stevenson, F.; Lau, R.; Murray, E.	Factors that influence the implementation of e-health: A systematic review of systematic reviews (an update)	*Implement Sci.*	0	2016	149	219.00
Naslund, J.; Marsch, L.; McHugo, G.; Bartels, S.	Emerging mHealth and eHealth interventions for serious mental illness: A review of the literature	*J. Ment. Health*	2.604	2015	156	216.00
[56] Torre-Díez, I.D.L.; Lopez-Coronado, M.; Vaca, C.; Saez Aguado, J.; De Castro, C.	Cost-utility and cost-effectiveness studies of telemedicine, electronic, and mobile health systems in the literature: A Systematic review	*Telemedicine and e-Health*	1.996	2015	150	210.00
Firth, J.; Torous, J.; Nicholas, J.; Carney, R.; Rosenbaum, S.; Sarris, J.	Can smartphone mental health interventions reduce symptoms of anxiety? A meta-analysis of randomized controlled trials	*J. Affect Disord.*	4.084	2017	123	203.00
Riley, R.D.; Ensor, J.; Snell, K.I.E.; Debray, T.P.A.; Altman, D.G., Moons, K.G.M.; Collins, G.S.	External validation of clinical prediction models using big datasets from e-health records or IPD meta-analysis: Opportunities and challenges	*BMJ—British Medical Journal*	27.604	2016	121	191.03
Poria, S.; Cambria, E.; Hussain, A.; Huang, G.B.	Towards an intelligent framework for multimodal affective data analysis	*Neural Networks*	5.785	2015	131	191.01
[74] Larsen, M.E.; Nicholas, J.; Christensen, H.	A systematic assessment of smartphone tools for suicide prevention	*Plos One*	2.776	2016	121	191.00
[64] Hollis, C.; Falconer, C.; Martin, J.; Whittington, C.; Stockton, S.; Glazebrook, C.; Davies, E.	Annual research review: digital health interventions for children and young people with mental health problems—a systematic and meta-review	*J. Child. Psychol. Psychiatry*	6.129	2017	109	189.01
Sultan, N.	Reflective thoughts on the potential and challenges of wearable technology for healthcare provision and medical education	*International Journal of Information Management*	5.063	2015	114	174.01
Aaronson, N.; Mattioli, V.; Minton, O.; Weis, J.; Johansen, C.; Dalton, S.; Verdonck-De Leeuw, I.; Stein, K.; Alfano, C.; Mehnert, A.; De Boer, A.; Van De Poll-Franse, L.	Beyond treatment—psychosocial and behavioural issues in cancer survivorship research and practice	*Eur. J. Cancer*	6.680	2014	123	173.01
Ben-Zeev, D.; Schueller, S.M.; Begale, M.; Duffecy, J.; Kane, J.M.; Mohr, D.C.	Strategies for mHealth research: lessons from 3 mobile intervention studies	*Adm. Policy. Ment. Health*	2.681	2015	111	171.00
[80] Moosavi, S.R.; Gia, T.N.; Rahmani, A.-M.; Nigussie, E.; Virtanen, S.; Isoaho, J.; Tenhunen, H.	SEA: A secure and efficient authentication and authorization architecture for IoT-based healthcare using smart gateways	*Procedia Comput. Sci.*	0	2015	111	171.00
Wagner, L.I.; Schink, J.; Bass, M.; Patel, S.; Diaz, M.V.; Rothrock, N.; Pearman, T.; Gershon, R.; Penedo, F.J.; Rosen, S.; Cella, D.	Bringing PROMIS to practice: Brief and precise symptom screening in ambulatory cancer care	*Cancer*	6.162	2015	110	170.01
Zhang, X.; Yu, P.; Yan, J.; Spil, I.T.A.M.	Using diffusion of innovation theory to understand the factors impacting patient acceptance and use of consumer e-health innovations: A case study in a primary care clinic	*BMC Health Serv. Res.*	1.932	2015	110	170.00
Ebert, D.; Berking, M.; Cuijpers, P.; Lehr, D.; Pörtner, M.; Baumeister, H.	Increasing the acceptance of internet-based mental health interventions in primary care patients with depressive symptoms. A randomized controlled trial	*J. Affect. Disord.*	4.084	2015	109	169.00
Clifton, L.; Clifton, D.; Pimentel, M.; Watkinson, P.; Tarassenko, L.	Predictive monitoring of mobile patients by combining clinical observations with data from wearable sensors	*IEEE J. Biomed. Health Inform.*	4.217	2014	118	168.00
McKay, F.H.; Cheng, C.; Wright, A.; Shill, J.; Stephens, H.; Uccellini, M.	Evaluating mobile phone applications for health behaviour change: A systematic review	*J. Telemed. Telecare*	2.229	2018	78	168.00
Olff, M.	Mobile mental health: A challenging research agenda	*Eur. J. Psychotraumatol*	3.020	2015	104	164.00
[45] Wentzel, J.; Van Der Vaart, R.; Bohlmeijer, E.T.; Van Gemert-Pijnen, J.E.W.C.	Mixing online and face-to-face therapy: How to benefit from blended care in mental health care	*JMIR Mental Health*	0	2016	93	163.00
[76] Grist, R.; Porter, J.; Stallard, P.	Mental health mobile apps for preadolescents and adolescents: A systematic review	*J. Med. Internet Res.*	4.945	2017	82	162.00
Yüksel, B.; Küpçü, A.; Özkasap, O.	Research issues for privacy and security of electronic health services	*Future Generation Computer Systems*	5.768	2017	77	157.01
Martin, S.; Feldman, D.; Blumenthal, R.; Jones, S.; Post, W.; McKibben, R.; Michos, E.; Ndumele, C.; Ratchford, E.; Coresh, J.; Blaha, M.	mActive: A randomized clinical trial of an automated mHealth intervention for physical activity promotion	*J. Am. Heart Assoc.*	4.660	2015	97	157.00
Martínez-Pérez, B.; Torre-Díez, I.D.L.; López-Coronado, M.; Sainz-De-Abajo, B.; Robles, M.; García-Gómez, J.	Mobile clinical decision support systems and applications: A literature and commercial review	*J. Med. Syst.*	2.415	2014	106	156.00
Tang, J.; Abraham, C.; Stamp, E.; Greaves, C.	How can weight-loss app designers’ best engage and support users? A qualitative investigation	*British Journal of Health Psychology*	2.472	2015	95	155.00
Alahäivälä, T.; Oinas-Kukkonen, H.	Understanding persuasion contexts in health gamification: A systematic analysis of gamified health behavior change support systems literature	*International Journal of Medical Informatics*	2.721	2016	83	153.00
Baker, T.; Gustafson, D.; Shah, D.	How can research keep up with eHealth? Ten strategies for increasing the timeliness and usefulness of Ehealth research	*J. Med. Internet Res.*	4.945	2014	102	152.00
Konstantinidis, E.I.; Billis, A.S.; Mouzakidis, C.A.; Zilidou, V.I.; Antoniou, P.E.; Bamidis, P.D.	Design, implementation, and wide pilot deployment of FitForAll: An easy to use exergaming platform improving physical fitness and life quality of senior citizens	*IEEE J. Biomed. Health Inform.*	4.217	2016	82	152.00
Pramana, G.; Parmanto, B.; Kendall, P.C.; Silk, J.S.	The SmartCAT: An m-Health platform for ecological momentary intervention in child anxiety treatment	*Telemedicine and e-Health*	1.996	2014	102	152.00
Watkins, I.; Xie, B.	eHealth literacy interventions for older adults: A systematic review of the literature	*J. Med. Internet Res.*	4.945	2014	101	151.00
Kaipio, J.; Lääveri, T.; Hyppönen, H.; Vainiomäki, S.; Reponen, J.; Kushniruk, A.; Borycki, E.; Vänskä, J.	Usability problems do not heal by themselves: National survey on physicians’ experiences with EHRs in Finland	*International Journal of Medical Informatics*	2.721	2017	70	150.00
Ahmad, M.; Amin, M.B.; Hussain, S.; Kang, B.H.; Cheong, T.; Lee, S.	Health Fog: A novel framework for health and wellness applications	*Journal of Supercomputing*	2.157	2016	80	150.00
Kim, H.; Xie, B.	Health literacy in the eHealth era: A systematic review of the literature	*Patient Educ. Couns.*	2.821	2017	68	148.00
Wicks, P.; Stamford, J.; Grootenhuis, M.; Haverman, L.; Ahmed, S.	Innovations in e-health	*Qual. Life Res.*	2.488	2014	97	147.00
Gómez, J.; Oviedo, B.; Zhuma, E.	Patient monitoring system based on Internet of Things	*Procedia Comput. Sci.*	0	2016	77	147.00
Cruz, J.; Brooks, D.; Marques, A.	Home telemonitoring in COPD: A systematic review of methodologies and patients’ adherence	*International Journal of Medical Informatics*	2.721	2014	96	146.00
Hubley, S.; Lynch, S.B.; Schneck, C.; Thomas, M.; Shore, J.	Review of key telepsychiatry outcomes	*World J. Psychiatry*	0	2016	71	141.00
Vegesna, A.; Tran, M.; Angelaccio, M.; Arcona, S.	Remote patient monitoring via non-invasive digital technologies: A systematic review	*Telemedicine and e-Health*	1.996	2017	60	140.00
[37] Brinkel, J.; Krämer, A.; Krumkamp, R.; May, J.; Fobil, J.	Mobile phone-based mHealth approaches for public health surveillance in sub-Saharan Africa: A systematic review	*Int. J. Environ. Res. Public Health*	2.468	2014	89	139.00
[57] Kleiboer, A.; Smit, J.; Bosmans, J.; Ruwaard, J.; Andersson, G.; Topooco, N.; Berger, T.; Krieger, T.; Botella, C.; Baños, R.; Chevreul, K.; Araya, R.; Cerga-Pashoja, A.; Cieślak, R.; Rogala, A.; Vis, C.; Draisma, S.; Schaik, A.; Kemmeren, L.; Ebert, D.; Berking, M.; Funk, B.; Cuijpers, P.; Riper, H.	European COMPARative Effectiveness research on blended Depression treatment versus treatment-as-usual (E-COMPARED): Study protocol for a randomized controlled, non-inferiority trial in eight European countries	*Trials*	1.975	2016	68	138.00
De Veer, A.J.E.; Peeters, J.M.; Brabers, A.E.M.; Schellevis, F.G.; Rademakers, J.J.D.J.M.; Francke, A.L.	Determinants of the intention to use e-health by community dwelling older people	*BMC Health Serv. Res.*	1.932	2015	78	138.00
[58] Provoost, S.; Lau, H.M.; Ruwaard, J.; Riper, H.	Embodied conversational agents in clinical psychology: A scoping review	*J. Med. Internet Res.*	4.945	2017	57	137.00
Schueller, S.; Tomasino, K.; Mohr, D.	Integrating human support into behavioral intervention technologies: The efficiency model of support	*Clinical Psychology: Science and Practice*	0	2017	57	137.00
Latulippe, K.; Hamel, C.; Giroux, D.	Social health inequalities and eHealth: A literature review with qualitative synthesis of theoretical and empirical studies	*J. Med. Internet Res.*	4.945	2017	56	136.00
Shaw, T.; McGregor, D.; Brunner, M.; Keep, M.; Janssen, A.; Barnet, S.	What is eHealth (6)? Development of a conceptual model for eHealth: Qualitative study with key informants	*J. Med. Internet Res.*	4.945	2017	56	136.00
[49] Pearce, C.; Bainbridge, M.	A personally controlled electronic health record for Australia	*J. Am. Med. Inform. Assoc.*	4.292	2014	86	136.00
Levack, W.; Weatherall, M.; Hay-Smith, E.; Dean, S.; Mcpherson, K.; Siegert, R.	Goal setting and strategies to enhance goal pursuit for adults with acquired disability participating in rehabilitation	*Cochrane Database of Systematic Reviews*	7.755	2015	75	135.01
[90] Brieger, D.; Amerena, J.; Attia, J.; Bajorek, B.; Chan, K.; Connell, C.; Freedman, B.; Ferguson, C.; Hall, T.; Haqqani, H.; Hendriks, J.; Hespe, C.; Hung, J.; Kalman, J.; Sanders, P.; Worthington, J.; Yan, T.; Zwar, N.	National Heart Foundation of Australia and Cardiac Society of Australia and New Zealand: Australian clinical guidelines for the diagnosis and management of atrial fibrillation 2018	*Med. J. Aust.*	0	2018	45	135.00
Brian, R.M.; Ben-Zeev, D.	Mobile health (mHealth) for mental health in Asia: Objectives, strategies, and limitations	*Asian Journal of Psychiatry*	1.932	2014	83	133.00
Ebert, D.D.; Cuijpers, P.; Munoz, R.F.; Baumeister, H.	Prevention of mental health disorders using internet- and mobile-based interventions: A narrative review and recommendations for future research	*Front Psychiatry*	3.161	2017	52	132.00
[55] Glasgow, R.E.; Phillips, S.M.; Sanchez, M.A.	Implementation science approaches for integrating eHealth research into practice and policy	*Int. J. Med. Inform.*	2.721	2014	82	132.00
[50] Bauermeister, J.; Pingel, E.; Jadwin-Cakmak, L.; Harper, G., Horvath, K.; Weiss, G.; Dittus, P.	Acceptability and preliminary efficacy of a tailored online HIV/STI testing intervention for young men who have sex with men: The Get Connected! program	*AIDS Behav.*	2.908	2015	70	130.00
Daniel, H.; Sulmasy, L.S.	Policy recommendations to guide the use of telemedicine in primary care settings: An American College of Physicians position paper	*Annals of Internal Medicine*	9.315	2015	68	128.01
Kazemi, D.M.; Borsari, B.; Levine, M.J., Li, S.; Lamberson, K.A.; Matta, L.A.	A systematic review of the mHealth interventions to prevent alcohol and substance abuse	*Journal of Health Communication*	1.773	2017	48	128.00
[62] Rotondi, A.J.; Eack, S.M.; Hanusa, B.H.; Spring, M.B.; Haas, G.L.	Critical design elements of e-health applications for users with severe mental illness: Singular focus, simple architecture, prominent contents, explicit navigation, and inclusive hyperlinks	*Schizophr. Bull.*	7.289	2015	67	127.01
Mohr, D.; Lyon, A.; Lattie, E.; Reddy, M.; Schueller, S.	Accelerating digital mental health research from early design and creation to successful implementation and sustainment	*J. Med. Internet Res.*	4.945	2017	47	127.00
Kumanyika, S.; Whitt-Glover, M.; Haire-Joshu, D.	What works for obesity prevention and treatment in black Americans? Research directions	*Obes. Rev.*	8.192	2014	75	125.01
Mooney, K.; Beck, S.; Wong, B.; Dunson, W.; Wujcik, D.; Whisenant, M.; Donaldson, G.	Automated home monitoring and management of patient-reported symptoms during chemotherapy: Results of the symptom care at home RCT	*Cancer Med.*	3.357	2017	45	125.00
Zhang, A.; Lin, X.	Towards secure and privacy-preserving data sharing in e-health systems via consortium blockchain	*J. Med. Syst.*	2.415	2018	34	124.00
Devlin, A.M.; McGee-Lennon, M.; O’Donnell, C.A.; Bouamrane, M.-M.; Agbakoba, R.; O’Connor, S.; Grieve, E.; Finch, T.; Wyke, S., Watson, N.; Browne, S.; Mair, F.S.; Team, D.E.	Delivering digital health and well-being at scale: Lessons learned during the implementation of the Dallas program in the United Kingdom	*J. Am. Med. Inform. Assoc.*	4.292	2016	53	123.00
Luna, D.; Almerares, A.; Mayan III, J.; De Quirós, F.; Otero, C.	Health informatics in developing countries: Going beyond pilot practices to sustainable implementations: A review of the current challenges	*Healthcare Informatics Research*	0	2014	73	123.00
Finnane, A.; Dallest, K.; Janda, M.; Soyer, H.P.	Teledermatology for the diagnosis and management of skin cancer: A systematic review	*JAMA Dermatol.*	7.995	2017	42	122.01
[63] Jackson, B.; Gray, K.; Knowlesd, S.; De Cruz, P.	EHealth technologies in inflammatory bowel disease: A systematic review	*Journal of Crohn’s and Colitis*	7.827	2016	52	122.01
Tinschert, P.; Jakob, R.; Barata, F.; Kramer, J.N.; Kowatsch, T.	The potential of mobile apps for improving asthma self-management: A review of publicly available and well-adopted asthma apps	*JMIR mHealth and uHealth*	4.301	2017	42	122.00
Hilty, D.; Crawford, A.; Teshima, J.; Chan, S.; Sunderji, N.; Yellowlees, P.; Kramer, G.; O’Neill, P.; Fore, C., Luo, J.; Li, S.T.	A framework for telepsychiatric training and e-health: Competency-based education, evaluation and implications	*International Review of Psychiatry*	2.991	2015	62	122.00
[73] Diviani, N.; Van Den Putte, B.; Meppelink, C.S.; Van Weert, J.C.	Exploring the role of health literacy in the evaluation of online health information: Insights from A mixed-methods study	*Patient Educ. Couns.*	2.821	2016	52	122.00
[6] Van Houwelingen, C.; Moerman, A.; Ettema, R.; Kort, H.; Ten Cate, O.	Competencies required for nursing telehealth activities: A Delphi-study	*Nurse Educ. Today*	2.442	2016	52	122.00
Molini-Avejonas, D.R.; Rondon-Melo, S.; Amato, C.A.D.L.H.; Samelli, A.G.	A systematic review of the use of telehealth in speech, language and hearing sciences	*J. Telemed Telecare*	2.229	2015	62	122.00
Chauhan, D.S.; Singh, A.K.; Kumar, B.; Saini, J.P.	Quantization based multiple medical information watermarking for secure e-health	*Multimedia Tools and Applications*	2.101	2019	22	122.00
Bradford, N.K.; Caffery, L.J.; Smith, A.C.	Telehealth services in rural and remote Australia: A systematic review of models of care and factors influencing success and sustainability	*Rural Remote Health*	0.985	2016	52	122.00
Townsend, A.; Leese, J.; Adam, P.; McDonald, M.; Li, L.C.; Kerr, S.; Backman, C.L.	eHealth, participatory medicine, and ethical care: A focus group study of patients’ and health care providers’ use of health-related internet information	*J. Med. Internet Res.*	4.945	2015	61	121.00
Tensen, E.; Van Der Heijden, J.; Jaspers, M.; Witkamp, L.	Two decades of teledermatology: Current status and integration in national healthcare systems	*Curr Dermatol Rep.*	0	2016	51	121.00
Andreassen, H.K.; Kjekshus, L.E.; Tjora, A.	Survival of the project: A case study of ICT innovation in health care	*Soc. Sci. Med.*	3.087	2015	60	120.00
[75] Malasinghe, L.P.; Ramzan, N.; Dahal, K.	Remote patient monitoring: A comprehensive study	*J. Ambient. Intell. Humaniz. Comput.*	1.910	2019	20	120.00
Shen, Q.; Liang, X.; Shen, X.S.; Lin, X.; Luo, H.Y.	Exploiting geo-distributed clouds for a e-health monitoring system with minimum service delay and privacy preservation	*IEEE J. Biomed. Health Inform.*	4.217	2014	69	119.00
Vinding, K.; Elsberg, H.; Thorkilgaard, T.; Belard, E.; Pedersen, N.; Elkjaer, M.; Marker, D.; Carlsen, K.; Burisch, J.; Munkholm, P.	Fecal calprotectin measured by patients at home using smartphones—A new clinical tool in monitoring patients with inflammatory bowel disease	*Inflammatory Bowel Diseases*	4.005	2016	49	119.00
Abdmeziem, M.R.; Tandjaoui, D.	An end-to-end secure key management protocol for e-health applications	*Computers & Electrical Engineering*	2.189	2015	59	119.00
[77] Lubberding, S.; Van Uden-Kraan, C.; Te Velde, E.; Cuijpers, P., Leemans, C.; Verdonck-de Leeuw, I.	Improving access to supportive cancer care through an eHealth application: A qualitative needs assessment among cancer survivors	*J. Clin. Nurs.*	1.757	2015	59	119.00
Leaman, R.; Khare, R.; Lu, Z.	Challenges in clinical natural language processing for automated disorder normalization	*J. Biomed. Inf.*	2.950	2015	58	118.00
Chung, S.Y.; Nahm, E.S.	Testing reliability and validity of the eHealth Literacy Scale (eHEALS) for older adults recruited online	*CIN—Computers Informatics Nursing*	1.029	2015	58	118.00
Iqbal, S.; Kiah, M.L.M.; Zaidan, A.A.; Zaidan, B.B.; Albahri, O.S.; Albahri, A.S.; Alsalem, M.A.	Real-time-based e-health systems: Design and implementation of a lightweight key management protocol for securing sensitive information of patients	*Health and Technology*	0	2019	18	118.00
East, M.L.; Havard, B.C.	Mental health mobile apps: From infusion to diffusion in the mental health social system	*JMIR Mental Health*	0	2015	58	118.00
[79] Domingues, M.; Alberto, N.; Leitão, C.; Tavares, C.; De Lima, E.; Radwan, A.; Sucasas, V.; Rodriguez, J.; Andre, P.; Antunes, P.	Insole optical fiber sensor architecture for remote gait analysis—an e-health solution	*IEEE Internet Things J.*	9.515	2019	17	117.01
[40] Baumel, A.; Faber, K.; Mathur, N.; Kane, J; Muench, F.	Enlight: A comprehensive quality and therapeutic potential evaluation tool for mobile and web-based eHealth interventions	*J. Med. Internet Res.*	4.945	2017	37	117.00
Zhang, L.; Zhu, S.; Tang, S.	Privacy protection for telecare medicine information systems using a chaotic map-based three-factor authenticated key agreement scheme	*IEEE J. Biomed. Health Inform.*	4.217	2017	37	117.00
[104] Zhou, J.; Cao, Z.; Dong, X.; Lin, X.	Security and privacy in cloud-assisted wireless wearable communications: Challenges, solutions and, future directions	*IEEE Wirel. Commun.*	3.546	2015	57	117.00
Maguire, R.; Ream, E.; Richardson, A.; Connaghan, J.; Johnston, B.; Kotronoulas, G.; Pedersen, V.; McPhelim, J.; Pattison, N.; Smith, A.; Webster, L.; Taylor, A.; Kearney, N.	Development of a novel remote patient monitoring system: The advanced symptom management system for radiotherapy to improve the symptom experience of patients with lung cancer receiving radiotherapy	*Cancer Nursing*	2.022	2015	57	117.00
Koivunen, M.; Saranto, K.	Nursing professionals’ experiences of the facilitators and barriers to the use of telehealth applications: A systematic review of qualitative studies	*Scand. J. Caring. Sci.*	1.642	2018	27	117.00
Thomas, J.; Bond, D.	Review of innovations in digital health technology to promote weight control	*Curr. Diab. Rep.*	0	2014	67	117.00
[7] Jhamb, M.; Cavanaugh, K.L.; Bian, A.; Chen, G.; Ikizler, T.A.; Unruh, M.L.; Abdel-Kader, K.	Disparities in electronic health record patient portal use in nephrology clinics	*Clin. J. Am. Soc. Nephrol.*	6.243	2015	56	116.01
[72] Mackert, M.; Champlin, S.E.; Holton, A.; Munoz, I.I.; Damasio, M.J.	eHealth and health literacy: A research methodology review	*J Comput. – Mediat. Commun.*	4.896	2014	66	116.00
Berrouiguet, S.; Perez-Rodriguez, M.M.; Larsen, M.; Baca-Garcia, E., Courtet, P.; Oquendo, M.	From eHealth to iHealth: Transition to participatory and personalized medicine in mental health	*J. Med. Internet Res.*	4.945	2018	25	115.00
Grönloh, C.; Myreteg, G.; Cajander, A.; Rexhepi, H.	“Why do they need to check me?” Patient participation through ehealth and the doctor-patient relationship: qualitative study	*J. Med. Internet Res.*	4.945	2018	25	115.00
Rouleau, G.; Gagnon, M.P.; Côté, J.; Payne-Gagnon, J.; Hudson, E.; Dubois, C.-A.	Impact of information and communication technologies on nursing care: Results of an overview of systematic reviews	*J. Med. Internet Res.*	4.945	2017	35	115.00
Lie, S.; Karlsen, B.; Oord, E.; Graue, M.; Oftedal, B.	Dropout from an eHealth intervention for adults with type 2 diabetes: A qualitative study	*J. Med. Internet Res.*	4.945	2017	35	115.00
Venkatesh, V.; Rai, A.; Sykes, T.; Aljafari, R.	Combating infant mortality in rural India: Evidence from a field study of Ehealth kiosk implementations	*Mis Q.*	4.373	2016	45	115.00
Christensen, H.; Batterham, P.; O’Dea, B.	E-health interventions for suicide prevention	*Int. J. Environ. Res. Public Health*	2.468	2014	65	115.00
Uscher-Pines, L.; Mehrotra, A.	Analysis of Teladoc use seems to indicate expanded access to care for patients without prior connection to a provider	*Health Aff. (Millwood)*	0	2014	65	115.00
[52] Wang, X.A.; Ma, J.; Xhafa, F.; Zhang, M.; Luo, X.	Cost-effective secure E-health cloud system using identity based cryptographic techniques	*Future Gener. Comp. Syst.*	5.768	2017	34	114.01
Meri, A.; Hasan, M.; Danaee, M.; Jaber, M.; Jarrar, M.; Safei, N.; Dauwed, M.; Abd, S.K.; Al-bsheish M.	Modelling the utilization of cloud health information systems in the Iraqi public healthcare sector	*Telematics and Informatics*	3.714	2019	14	114.00
Handayani, P.W.; Hidayanto, A.N.; Pinem, A.A.; Hapsari, I.C.; Sandhyaduhita, P.I.; Budi, I.	Acceptance model of a hospital information system	*International Journal of Medical Informatics*	2.721	2017	34	114.00
Kampmeijer, R.; Pavlova, M.; Tambor, M.; Golinowska, S.; Groot, W.	The use of e-health and m-health tools in health promotion and primary prevention among older adults: A systematic literature review	*BMC Health Serv Res*	1.932	2016	44	114.00
[53] Romanou, A.	The necessity of the implementation of Privacy by Design in sectors where data protection concerns arise	*Computer Law & Security Review*	1.552	2018	24	114.00
Hoque, M.R.; Bao, Y.; Sorwarb, G.	Investigating factors influencing the adoption of e-health in developing countries: A patient’s perspective	*Inform. Health Soc. Care*	1.218	2017	34	114.00
Zhang, L.; Zhang, Y.; Tang, S.; Luo, H.	Privacy protection for e-health systems by means of dynamic authentication and three-factor key agreement	*IEEE Trans. Ind. Electron.*	7.503	2018	23	113.01
[42] García, L.; Tomás, J.; Parra, L.; Lloret, J.	An m-health application for cerebral stroke detection and monitoring using cloud services	*Inf. Int. J. Manag.*	5.063	2019	13	113.01
Greenhalgh, T.; Stones, R.; Swinglehurst, D.	Choose and book: A sociological analysis of “resistance” to an expert system	*Soc. Sci. Med.*	3.087	2014	63	113.00
Giunti, G.; Giunta, D.; Guisado-Fernandez, E.; Bender, J.; Fernandez-Luque, L.	A biopsy of breast cancer mobile applications: State of the practice review	*International Journal of Medical Informatics*	2.721	2018	23	113.00
Bujnowska-Fedak, M.M.; Pirogowicz, I.	Support for e-health services among elderly primary care patients	*Telemedicine and e-Health*	1.996	2014	63	113.00
Meurk, C.; Leung, J.; Hall, W.; Head, B.W.; Whiteford, H.	Establishing and governing e-mental health care in Australia: A systematic review of challenges and a call for policy-focussed research	*J. Med. Internet Res.*	4.945	2016	42	112.00
Ma, J.; Zhang, T.; Dong, M.	A novel ECG data compression method using adaptive fourier decomposition with security guarantee in e-health applications	*IEEE J. Biomed. Health Inform.*	4.217	2015	52	112.00
[61] Pedersen, S.J.; Cooley, P.D.; Mainsbridge, C.	An e-health intervention designed to increase workday energy expenditure by reducing prolonged occupational sitting habits	*Work—A Journal of Prevention Assessment & Rehabilitation*	1.009	2014	62	112.00
Quanbeck, A.; Gustafson, D.H.; Marsch, L.A.; Chih, M.Y.; Kornfield, R.; McTavish, F.; Johnson, R.; Brown, R.T.; Mares, M.L.; Shah, D.V.	Implementing a mobile health system to integrate the treatment of addiction into primary care: A hybrid implementation-effectiveness study	*J. Med. Internet Res.*	4.945	2018	21	111.00
Grünloh, C.; Cajander, A; Myreteg, G.	The record is our work tool-physicians’ framing of a patient portal in Sweden	*J. Med. Internet Res.*	4.945	2016	41	111.00
Sims, J.	Communities of practice: telemedicine and online medical communities	*Technol. Forecast. Soc. Change*	3.815	2018	21	111.00
[41] Litchman, M.; Rothwell, E.; Edelman, L.	The diabetes online community: older adults supporting self-care through peer health	*Patient Educ. Couns.*	2.821	2018	21	111.00
[70] Paige, S.; Krieger, J.; Stellefson, M.; Alber, J.	eHealth literacy in chronic disease patients: An item response theory analysis of the eHealth literacy scale (eHEALS)	*Patient Educ. Couns.*	2.821	2017	31	111.00
Vijayakumar, V.; Priyan, M.K.; Ushadevi, G.; Varatharajan, R.; Manogaran, G.; Tarare, P.V.	E-health cloud security using timing enabled proxy re-encryption	*Mobile Networks & Applications*	2.390	2019	11	111.00
Liddy, C.; Moroz, I.; Mihan, A.; Nawar, N.; Keely, E.	A systematic review of asynchronous, provider-to-provider, electronic consultation services to improve access to specialty care available worldwide	*Telemedicine and e-Health*	1.996	2019	11	111.00
Heisler, M.; Choi, H.; Palmisano, G.; Mase, R.; Richardson, C.; Fagerlin, A.; Montori, V.; Spencer, M.; An, L.	Comparison of community health worker-led diabetes medication decision-making support for low-income Latino and African American adults with diabetes using e-health tools versus print materials	*Annals of Internal Medicine*	19.315	2014	60	110.02
Chouvarda, I.G.; Goulis, D.G.; Lambrinoudaki, I.; Maglaveras, N.	Connected health and integrated care: Toward new models for chronic disease management	*Maturitas*	3.654	2015	50	110.00
Ferreri, F.; Bourla, A.; Mouchabac, S.; Karila, L.	e-Addictology: An overview of new technologies for assessing and intervening in addictive behaviors	*Front. Psychiatry*	3.162	2018	20	110.00
Lee, E.W.; Denison, F.C.; Hor, K.; Reynolds, R.M.	Web-based interventions for prevention and treatment of perinatal mood disorders: A systematic review	*BMC Pregnancy and Childbirth*	2.413	2016	40	110.00
Pietro, C.D.; Francetic, I.	E-health in Switzerland: the laborious adoption of the federal law on electronic health records (EHR) and health information exchange (HIE) networks	*Health Policy*	2.075	2018	20	110.00
Bol, N.; Helberger, N.; Weert, J.C.M.	Differences in mobile health app use: A source of new digital inequalities?	*Information Society*	1.860	2018	20	110.00
[99] Minoli, D.; Occhiogrosso, B.	Blockchain mechanisms for IoT security	*Internet of Things*	0	2018	20	110.00
Makhdoom, I.; Abolhasan, M.; Lipman, J.; Liu, R.; Ni, W.	Anatomy of threats to the Internet of Things	*IEEE Communications Surveys and Tutorials*	22.973	2019	9	109.02
Pussewalage, H.S.G.; Oleshchuk, V.A.	Privacy preserving mechanisms for enforcing security and privacy requirements in e-health solutions	*International Journal of Information Management*	5.063	2016	39	109.01
Dimidjian, S.; Beck, A.; Felder, J.N.; Boggs, J.M.; Gallop, R.; Segal, Z.V.	Web-based mindfulness-based cognitive therapy for reducing residual depressive symptoms: An open trial and quasi-experimental comparison to propensity score matched controls	*Behaviour Research and Therapy*	4.309	2014	59	109.00
Pedersen, N.; Thielsen, P.; Martinsen, L.; Bennedsen, M.; Haaber, A.; Langholz, E.; Végh, Z.; Duricova, D.; Jess, T.; Bell, S.; Burisch, J.; Munkholm, P.	EHealth: Individualization of mesalazine treatment through a self-managed web-based solution in mild-to-moderate ulcerative colitis	*Inflammatory Bowel Diseases*	4.005	2014	59	109.00
Aardoom, J.J.; Dingemans, A.E.; Van Furth, E.F.	E-health interventions for eating disorders: Emerging findings, issues, and opportunities	*Curr. Psychiatry Rep.*	3.816	2016	39	109.00
Machado, G.; Pinheiro, M.; Lee, H.; Ahmed, O.; Hendrick, P., Williams, C.; Kamper, S.	Smartphone apps for the self-management of low back pain: A systematic review	*Best Practice & Research Clinical Rheumatology*	3.016	2016	39	109.00
Slev, V.N.; Mistiaen, P.; Pasman, H.R.W.; Verdonck-de Leeuw, I.M.; van Uden-Kraan, C.F.; Francke, A.L.	Effects of eHealth for patients and informal caregivers confronted with cancer: A meta-review	*International Journal of Medical Informatics*	2.721	2016	39	109.00
Hossain, N.; Yokota, F.; Sultana, N.; Ahmed, A.	Factors influencing rural end-users’ acceptance of e-health in developing countries: A study on portable health clinic in Bangladesh	*Telemedicine and e-Health*	1.996	2019	9	109.00
López-Jaquero, V.; Montero, F.; Teruel, M.	Influence awareness: Considering motivation in computer-assisted rehabilitation	*J. Ambient Intell. Humaniz. Comput.*	1.910	2019	9	109.00
Robertson, N.; Polonsky, M.; McQuilken, L.	Are my symptoms serious Dr Google? A resource-based typology of value co-destruction in online self-diagnosis	*Australasian Marketing Journal (AMJ)*	0	2014	59	109.00
Li, F.; Li, Z.; Han, W.; Wu, T.; Chen, L.; Guo, Y.; Chen, J.	Cyberspace-oriented access control: A cyberspace characteristics-based model and its policies	*IEEE Internet Things J.*	9.516	2019	8	108.01
Aghili, S.F.; Mala, H.; Shojafar, M.; Peris-Lopez, P.	LACO: Lightweight three-factor authentication, access control and ownership transfer scheme for e-health systems in IoT	*Future Generation Computer Systems*	5.768	2019	8	108.01
Yang, Y.; Zheng, X.; Liu, X.; Zhong, S.; Chang, V.	Cross-domain dynamic anonymous authenticated group key management with symptom-matching for e-health social system	*Future Generation Computer Systems*	5.768	2018	18	108.01
Vitacca, M.; Montini, A.; Comini, L.	How will telemedicine change clinical practice in chronic obstructive pulmonary disease?	*Ther. Adv. Respir. Dis.*	3.286	2018	18	108.00
[71] Holmberg, C.; Berg, C.; Dahlgren, J.; Lissner, L.; Chaplin, J.E.	Health literacy in a complex digital media landscape: pediatric obesity patients’ experiences with online weight, food, and health information	*Health Informatics J.*	2.297	2019	8	108.00
Irving, M.; Stewart, R.; Spallek, H.; Blinkhorn, A.	Using teledentistry in clinical practice as an enabler to improve access to clinical care: A qualitative systematic review	*J. Telemed. Telecare*	2.229	2018	18	108.00
Saba, T.; Khan, S.U.; Islam, N.; Abbas, N.; Rehman, A.; Javaid, N.; Anjum, A.	Cloud-based decision support system for the detection and classification of malignant cells in breast cancer using breast cytology images	*Microsc. Res. Tech.*	1.327	2019	8	108.00
[8] De Grood, C.; Raissi, A.; Kwon, Y.; Santana, M.J.	Adoption of e-health technology by physicians: A scoping review	*J. Multidiscip. Healthc.*	0	2016	38	108.00
Hamza, R.; Yan, Z.; Muhammad, K.; Bellavista, P.; Titouna, F.	A privacy-preserving cryptosystem for IoT E-healthcare	*Information Sciences*	5.524	2019	7	107.01
Greenwood, D.; Blozis, S.; Young, H.; Nesbitt, T.; Quinn, C.	Overcoming clinical inertia: A randomized clinical trial of a telehealth remote monitoring intervention using paired glucose testing in adults with type 2 diabetes	*J. Med. Internet Res.*	4.945	2015	47	107.00
Cozza, M.; Crevani, L.; Hallin, A.; Schaeffer, J.	Future ageing: Welfare technology practices for our future older selves	*Futures*	2.214	2019	7	107.00
Amato, F.; Moscato, F.	A model driven approach to data privacy verification in e-health systems	*Transactions on Data Privacy*	0	2015	47	107.00
[10] Swinkels, I.C.S.; Huygens, M.W.J.; Schoenmakers, T.M.; Nijeweme-D’Hollosy, W.O.; Van Velsen, L.; Vermeulen, J.; Schoone-Harmsen, M.; Jansen, Y.J.F.M.; Van Schayck, O.C.P.; Friele, R.; Witte, L. de	Lessons learned from a living lab on the broad adoption of eHealth in primary health care	*J. Med. Internet Res.*	4.945	2018	16	106.00
Volker, D.; Zijlstra-Vlasveld, M.C.; Anema, J.R.; Beekman, A.T.F.; Brouwers, E.P.M.; Emons, W.H.M.; Van Lomwel, A.G.C.; Van Der Feltz-Cornelis, C.M.	Effectiveness of a blended web-based intervention on return to work for sick-listed employees with common mental disorders: Results of a cluster randomized controlled trial	*J. Med. Internet Res.*	4.945	2015	46	106.00
Børøsund, E.; Cvancarova, M.; Moore, S.; Ekstedt, M.; Ruland, C.	Comparing effects in regular practice of e-communication and web-based self-management support among breast cancer patients: Preliminary results from a randomized controlled trial	*J. Med. Internet Res.*	4.945	2014	56	106.00
Noonan, V.K.; Lyddiatt, A.; Ware, P.; Jaglal, S.B.; Riopelle, R.J.; Bingham III, C.O.; Figueiredo, S.; Sawatzky, R.; Santana, M.; Bartlett, S.J.;Ahmed, S.	Montreal Accord on Patient-Reported Outcomes (PROs) use series—Paper 3: Patient-reported outcomes can facilitate shared decision-making and guide self-management	*J. Clin. Epidemiol.*	4.65	2017	26	106.00
Nguyen, D.C.; Pathirana, P.N.; Ding, M.; Seneviratne, A.	Blockchain for secure EHRs sharing of mobile cloud based e-health system	*Access, IEEE*	4.098	2019	6	106.00
[97] Wang, P.; Ye, F.; Chen, X.; Qian, Y.	Datanet: Deep learning based encrypted network traffic classification in SDN Home Gateway	*Access, IEEE*	4.098	2018	16	106.00
Steins, D.; Dawes, H.; Esser, P.; Collett, J.	Wearable accelerometry-based technology capable of assessing functional activities in neurological populations in community settings: A systematic review	*J. Neuroeng. Rehabil.*	3.582	2014	56	106.00
[54] Wass, S.; Vimarlund, V.; Ros, A.	Exploring patients’ perceptions of accessing electronic health records: Innovation in healthcare	*Health Informatics J.*	2.297	2019	6	106.00
Hall, A.K.; Bernhardt, J.M.; Dodd, V.; Vollrath, M.W.	The digital health divide: Evaluating online health information access and use among older adults	*Health Education & Behavior*	2.190	2015	46	106.00
Girault, A.; Ferrua, M.; Lalloué, B.; Sicotte, C.; Fourcade, A.; Yatim, F.; Hébert, G.; Palma, M.D.; Minvielle, E.	Internet-based technologies to improve cancer care coordination: Current use and attitudes among cancer patients	*Eur. J. Cancer*	6.68	2015	45	105.01
Khan, S.U.; Islam, N.; Jan, Z.; Din, I.U.; Khan, A.; Faheem, Y.	An e-health care services framework for the detection and classification of breast cancer in breast cytology images as an IoMT application	*Future Generation Computer Systems*	5.768	2019	5	105.01
Hsu, W.; Chiang, C.; Yang, S.	The effect of individual factors on health behaviors among college students: The mediating effects of eHealth literacy	*J. Med. Internet Res.*	4.945	2014	55	105.00
[18] Parikh, S.V.; Huniewicz, P.	E-health: An overview of the uses of the internet, social media, apps, and websites for mood disorders	*Curr. Opin. Psychiatry*	4.483	2015	45	105.00
Wazid, M.; Das, A.K.; Kumar, N.; Odelu, V.; Reddy, A.G.; Parks, K.; Parks, Y.	Design of lightweight authentication and key agreement protocol for vehicular ad hoc networks	*Access, IEEE*	4.098	2017	25	105.00
Tosi, J.; Taffoni, F.; Santacatterina, M.; Sannino, R.; Formica, D.	Performance evaluation of bluetooth low energy: A systematic review	*Sensors*	3.031	2017	25	105.00
Wagenaar, K.P.; Broekhuizen, B.D.L.; Jaarsma, T.; Kok, I.; Mosterd, A.; Willems, F.F.; Linssen, G.C.M.; Agema, W.R.P.; Anneveldt, S.; Lucas, C.M.H.B.; Mannaerts, H.F.J.; Wajon, E.M.C.J.; Dickstein, K.; Cramer, M.J.; Landman, M.A.J.; Hoes, A.W.; Rutten, F.H.	Effectiveness of the European Society of Cardiology/Heart Failure Association website “heartfailurematters.org” and an e-health adjusted care pathway in patients with stable heart failure: Results of the “e-Vita HF” randomized controlled trial	*Eur. J. Heart Fail.*	2.784	2019	5	105.00
Wernhart, A.; Gahbauer, S.; Haluza, D.	eHealth and telemedicine: Practices and beliefs among healthcare professionals and medical students at a medical university	*Plos One*	2.776	2019	5	105.00
Maramba, I.; Chatterjee, A.; Newman, C.	Methods of usability testing in the development of eHealth applications: A scoping review	*International Journal of Medical Informatics*	2.721	2019	5	105.00
Daher, J.; Vijh, R.; Linthwaite, B.; Dave, S.; Kim, J.; Dheda, K.; Peter, T.; Pai, N.	Do digital innovations for HIV and sexually transmitted infections work? Results from a systematic review (1996–2017)	*BMJ Open*	2.376	2017	25	105.00
Bucci, S.; Schwannauer, M.; Berry, N.	The digital revolution and its impact on mental health care	*Psychol Psychother*	2.244	2019	5	105.00
Oueida, S.; Aloqaily, M.; Ionescu, S.	A smart healthcare reward model for resource allocation in smart city	*Multimedia Tools and Applications*	2.101	2019	5	105.00
Zhao, X.; Yang, B.; Wong, C.-W.	Analyzing trend for U.S. Immigrants’ e-health engagement from 2008 to 2013	*Health Communication*	1.846	2019	5	105.00
Keasberry, J.; Scott, I.A.; Sullivan, C.; Staib, A.; Ashby, R.	Going digital: A narrative overview of the clinical and organisational impacts of eHealth technologies in hospital practice	*Australian Health Review*	1.228	2017	25	105.00
Helle, C.; Hillesund, E.; Wills, A.; Øverby, N.	Evaluation of an eHealth intervention aiming to promote healthy food habits from infancy—the Norwegian randomized controlled trial Early Food for Future Health	*IJBNPA*	6.037	2019	4	104.01
Hammersley, M.; Okely, A.; Batterham, M.; Jones, R.	An internet-based childhood obesity prevention program (TIMe2bhealthy) for parents of preschool-aged children: randomized controlled trial	*J. Med. Internet Res.*	4.945	2019	4	104.00
Adam, M.; McMahon, S.; Prober, C.; Bärnighausen, T.	Human-centered design of video-based health education: An iterative, collaborative, community-based approach	*J. Med. Internet Res.*	4.945	2019	4	104.00
MacDonald, G.; Townsend, A.; Adam, P.; Li, L.; Kerr, S.; McDonald, M.; Backman, C.	eHealth technologies, multimorbidity, and the office visit: Qualitative interview study on the perspectives of physicians and nurses	*J. Med. Internet Res.*	4.945	2018	14	104.00
Orchard, J.; Neubeck, L.; Freedman, B.; Li, J.; Webster, R.; Zwar, N.; Gallagher, R.; Ferguson, C.; Lowres, N.	eHealth tools to provide structured assistance for atrial fibrillation screening, management, and guideline-recommended therapy in metropolitan general practice: The AF-SMART study	*J. Am. Heart Assoc.*	4.660	2019	4	104.00
Alwashmi, M.F.; Hawboldt, J.; Davis, E.; Fetters, M.D.	The iterative convergent design for mobile health usability testing: Mixed-methods approach	*JMIR mHealth and uHealth*	4.301	2019	4	104.00
Goetz, M.; Muller, M.; Matthies, L.M.; Hansen, J.; Doster, A.; Szabo, A.; Pauluschke-Froehlich, J.; Abele, H., Sohn, C.; Wallwiener, M.; Wallwiener, S.	Perceptions of patient engagement applications during pregnancy: A qualitative assessment of the patient’s perspective	*JMIR mHealth and uHealth*	4.301	2017	24	104.00
Warrington, L.; Absolom, K.; Velikova, G.	Integrated care pathways for cancer survivors—a role for patient-reported outcome measures and health informatics	*Acta Oncologica*	3.298	2015	44	104.00
Gossec, L.; Molto, A.; Romand, X.; Puyraimond-Zemmour, D.; Lavielle, M.; Beauvais, C.; Senbel, E.; Flipo, R.M.; Pouplin, S.; Richez, C.; Saraux, A.; Mézières, M.; Gutermann, L., Gaudin, P.; Wendling, D.; Dougados, M.	Recommendations for the assessment and optimization of adherence to disease-modifying drugs in chronic inflammatory rheumatic diseases: A process based on literature reviews and expert consensus	*Joint Bone Spine*	3.278	2019	4	104.00
Kitsios, F.; Stefanakakis, S.; Kamariotou, M.; Dermentzoglou, L.	E-service evaluation: User satisfaction measurement and implications in health sector	*Computer Standards & Interfaces*	2.441	2019	4	104.00
Lindner, P.; Miloff, A.; Zetterlund, E.; Reuterskiold, L.; Andersson, G.; Carlbring, P.	Attitudes toward and familiarity with virtual reality therapy among practicing cognitive behavior therapists: A cross-sectional survey study in the era of consumer VR platforms	*Frontiers in Psychology*	2.129	2019	4	104.00
Cho, Y.M.; Lee, S.; Islam, S.; Kim, S.Y.	Theories applied to m-health interventions for behavior change in low- and middle-income countries: A systematic review	*Telemedicine and e-Health*	1.996	2018	14	104.00
Terrasse, M., Gorin, M.; Sisti, D.	Social media, e-health, and medical ethics	*Hastings Center Report*	1.728	2019	4	104.00
Öberg, U.; Orre, C.; Isaksson, U.; Schimmer, R.; Larsson, H.; Hörnsten, Å.	Swedish primary healthcare nurses’ perceptions of using digital eHealth services in support of patient self-management	*Scand. J. Caring Sci.*	1.642	2018	14	104.00
Razmak, J.; Belanger, C.	Using the technology acceptance model to predict patient attitude toward personal health records in regional communities	*Information Technology & People*	1.263	2018	14	104.00
[96] Thomas, N.; Bless, J.J.; Alderson-Day, B.; Bell, I.H.; Cella, M.; Craig, T.; Delespaul, P.; Hugdahl, K.; Laloyaux, J.; Larøi, F.; Lincoln, T.M.; Schlier, B.; Urwyler, P.; van den Berg, D.; Jardri, R.	Potential applications of digital technology in assessment, treatment, and self-help for hallucinations	*Schizophr. Bull.*	7.289	2019	3	103.01
Willmott, T.; Pang, B.; Rundle-Thiele, S.; Badejo, A.	Weight management in young adults: Systematic review of electronic health intervention components and outcomes	*J. Med. Internet Res.*	4.945	2019	3	103.00
Van Den Heuvel, J.; Groenhof, T.; Veerbeek, J.; Van Solinge, W.; Lely, A.; Franx, A.; Bekker, M.	eHealth as the next-generation perinatal care: An overview of the literature	*J. Med. Internet Res.*	4.945	2018	13	103.00
Chenthara, S.; Ahmed, K.; Wang, H.; Whittaker, F.	Security and privacy-preserving challenges of e-health solutions in cloud computing	*Access, IEEE*	4.098	2019	3	103.00
[94] Vloothuis, J.D.M.; Mulder, M.; Nijland, R.H.M.; Goedhart, Q.S.; Konijnenbelt, M.; Mulder, H.; Hertogh, C.M.P.M.; van Tulder, M.; van Wegen, E.E.H.; Kwakkel, G.	Caregiver-mediated exercises with e-health support for early supported discharge after stroke (CARE4STROKE): A randomized controlled trial	*Plos One*	2.776	2019	3	103.00
Cheatle, M.	Biopsychosocial approach to assessing and managing patients with chronic pain	*Medical Clinics of North America*	2.716	2016	33	103.00
Ahmed, B.; Dannhauser, T.; Philip, N.	A systematic review of reviews to identify key research opportunities within the field of eHealth implementation	*J. Telemed. Telecare*	2.229	2019	3	103.00
[48] Schettini, P.; Shah, K.; O’Leary, C.; Patel, M.; Anderson, J.; Cho, A.; Long, A.; Bosworth, H.; Cameron, C.	Keeping care connected: E-Consultation program improves access to nephrology care	*J. Telemed. Telecare*	2.229	2019	3	103.00
Karekla, M.; Kasinopoulos, O.; Neto, D.; Ebert, D.; Van Daele, T.; Nordgreen, T.; Höfer, S.; Oeverland, S.; Jensen, K.	Best practices and recommendations for digital interventions to improve engagement and adherence in chronic illness sufferers	*European Psychologist*	2.167	2019	3	103.00
De Rosis, S.; Barsanti, S.	Patient satisfaction, e-health and the evolution of the patient-general practitioner relationship: Evidence from an Italian survey	*Health Policy*	2.075	2016	33	103.00
Zonneveld, M.; Patomella, A.H.; Asaba, E.; Guidetti, S.	The use of information and communication technology in healthcare to improve participation in everyday life: A scoping review	*Disability and rehabilitation*	2.054	2019	3	103.00
Leung, L.; Chen, C.	E-health/m-health adoption and lifestyle improvements: Exploring the roles of technology readiness, the expectation-confirmation model, and health-related information activities	*Telecomm Policy*	2.000	2019	3	103.00
Kerst, A.; Zielasek, Jü.; Gaebel, W.	Smartphone applications for depression: A systematic literature review and a survey of health care professionals&039; attitudes towards their use in clinical practice	*Eur. Arch. Psychiatr. Clin. Neurosci.*	0	2019	3	103.00
Kubendiran, M.; Singh, S.; Sangaiah, A.K.	Enhanced security framework for e-health systems using blockchain	*Journal of Information Processing Systems*	0	2019	3	103.00
Celesti, A.; Fazio, M.; Márquez, F.; Glikson, A.; Mauwa, H.; Bagula, A., Celesti, F.; Villari, M.	How to develop IoT cloud e-health systems based on fiware: A lesson learnt	*Journal of Sensor and Actuator Networks*	0	2019	3	103.00
Alvarez, C.; Fedock, G.	Addressing intimate partner violence with Latina women: A call for research	*Trauma Violence Abuse*	0	2018	13	103.00
Van Der Meij, E.; Anema, J.R.; Leclercq, W.K.G.; Bongers, M.Y.; Consten, E.C.J.; Koops, S.E.S.; Van De Ven, P.M.; Terwee, C.B.; Van Dongen, J.M.; Schaafsma, F.G.; Meijerink, W.J.H.J.; Bonjer, H.J.; Huirne, J.A.F.	Personalised perioperative care by e-health after intermediate-grade abdominal surgery: A multicentre, single-blind, randomised, placebo-controlled trial	*Lancet*	59.102	2018	12	102.06
Islam, N.; Faheem, Y.; Din, I.U.; Talha, M.; Guizani, M.; Khalil, M.	A blockchain-based fog computing framework for activity recognition as an application to e-healthcare services	*Future Generation Computer Systems*	5.768	2019	2	102.01
Peddi, V.B.; Kuhad, P.; Yassine, A.; Pouladzadeh, P.; Shirmohammadi, S.; Shirehjini, A.A.N.	An intelligent cloud-based data processing broker for mobile e-health multimedia applications	*Future Generation Computer Systems*	5.768	2017	22	102.01
Chen, Y.; Crespi, N.; Ortiz, A.M.; Shu, L.	Reality mining: A prediction algorithm for disease dynamics based on mobile Big Data	*Information Sciences*	5.524	2017	22	102.01
Shaw, T.; Janssen, A.; Crampton, R.; O’Leary, F.; Hoyle, P.; Jones, A.; Shetty, A.; Gunja, N.; Ritchie, A.; Spallek, H.; Solman, A.; Kay, J., Makeham, M.; Harnett, P.	Attitudes of health professionals to using routinely collected clinical data for performance feedback and personalised professional development	*Med. J. Aust.*	5.332	2019	2	102.01
[89] Sakakibara, B.; Chakrabarti, S.; Krahn, A.; Mackay, M.; Sedlak, T.; Singer, J.; Whitehurst, D.; Lear, S.	Delivery of peer support through a self-management mHealth intervention (healing circles) in patients with cardiovascular disease: Protocol for a randomized controlled trial	*J. Med. Internet Res.*	4.945	2019	2	102.00
Vis, C.; Ruwaard, J.; Finch, T.; Rapley, T.; De Beurs, D.; Van Stel, H.; Van Lettow, B.; Mol, M.; Kleiboer, A.; Riper, H.; Smit, J.	Toward an objective assessment of implementation processes for innovations in health care: Psychometric evaluation of the normalization Measure Development (NOMAD) questionnaire among mental health care professionals	*J. Med. Internet Res.*	4.945	2019	2	102.00
Wu, B.	Patient continued use of online health care communities: Web mining of patient-doctor communication	*J. Med. Internet Res.*	4.945	2018	12	102.00
Hossain, M.; Poon, C.; Dong, Y.; Lo, I.; Cheng, J.	Development of social sustainability assessment method and a comparative case study on assessing recycled construction materials	*International Journal of Life Cycle Assessment*	4.868	2018	12	102.00
Barteit, S.; Neuhann, F.; Barnighausen, T.; Luders, S.; Malunga, G.; Chileshe, G.; Marimo, C.; Jahn, A.	Perspectives of nonphysician clinical students and medical lecturers on tablet-based health care practice support for medical education in Zambia, Africa: Qualitative study	*JMIR mHealth and uHealth*	4.301	2019	2	102.00
Antonio, M.; Petrovskaya, O.; Lau, F.	Is research on patient portals attuned to health equity? A scoping review	*J. Am. Med. Inform. Assoc.*	4.292	2019	2	102.00
Tursunbayeva, A.; Bunduchi, R.; Franco, M.; Pagliari, C.	Human resource information systems in health care: A systematic evidence review	*J. Am. Med. Inform. Assoc.*	4.292	2017	22	102.00
Odelu, V.; Saha, S.; Prasath, R.; Sadineni, L.; Conti, M.; Jo, M.	Efficient privacy preserving device authentication in WBANs for industrial e-health applications	*Computers & Security*	3.062	2019	2	102.00
Devan, H.; Perry, M.A.; Van Hattem, A.; Thurlow, G.; Shepherd, S., Muchemwa, C.; Grainger, R.	Do pain management websites foster self-management support for people with persistent pain? A scoping review	*Patient Educ. Couns.*	2.821	2019	2	102.00
Carcone, A.; Hasan, M.; Alexander, G.; Dong, M.; Eggly, S.; Hartlieb, K.; Naar, S.; MacDonell, K.; Kotov, A.	Developing machine learning models for behavioral coding	*J. Pediatr. Psychol.*	2.670	2019	2	102.00
Lo Presti, L.; Testa, M.; Marino, V.; Singer, P.	Engagement in healthcare systems: Adopting digital tools for a sustainable approach	*Sustainability*	2.592	2019	2	102.00
Ross, J.; Stevenson, F.; Lau, R.; Murray, E.	Exploring the challenges of implementing e-health: A protocol for an update of a systematic review of reviews	*BMJ Open*	2.376	2015	42	102.00
Heathcote, L.; Pate, J.; Park, A.; Leake, H.; Lorimer Moseley, G.; Kronman, C.; Fischer, M.; Timmers, I.; Simons, L.	Pain neuroscience education on YouTube	*PEERJ*	2.353	2019	2	102.00
Azeez, N.A.; Der Vyver, C.	Security and privacy issues in e-health cloud-based system: A comprehensive content analysis	*Egyptian Informatics Journal*	2.306	2019	2	102.00
Prado, G.; Estrada, Y.; Rojas, L.M.; Bahamon, M.; Pantin, H.; Nagarsheth, M.; Gwynn, L.; Ofir, A.Y.; Forster, L.Q., Torres, N.; Brown, C.H.	Rationale and design for eHealth Familias Unidas Primary Care: A drug use, sexual risk behavior, and STI preventive intervention for Hispanic youth in pediatric primary care clinics	*Contemp. Clin. Trials*	2.280	2019	2	102.00
Guertler, D.; Moehring, A.; Krause, K.; Eck, S.; Batra, A.; Chenot, J.-F.; Freyer-Adam, J.; Ulbricht, S.; Rumpf, H.-J.; Bischof, G.; John, U.; Meyer, C.	Proactive multipurpose health risk screening in health care settings: Methods, design, and reach	*Int. J. Method Psych.*	2.276	2019	2	102.00
Possemato, K.; Johnson, E.M.; Emery, J.B.; Wade, M.; Acosta, M.C.; Marsch, L.A.; Rosenblum, A.; Maisto, S.A.	A pilot study comparing peer supported web-based CBT to self-managed web CBT for primary care veterans with PTSD and hazardous alcohol use	*Psychiatric Rehabilitation Journal*	2.270	2019	2	102.00
Hossain, N.; Sampa, M.B.; Yokota, F.; Fukuda, A.; Ahmed, A.	Factors affecting rural patients’ primary compliance with e-prescription: A developing country perspective	*Telemedicine and e-Health*	1.996	2019	2	102.00
AlBar, A.M.; Hoque, M.R.	Patient acceptance of e-health services in Saudi Arabia: An integrative perspective	*Telemedicine and e-Health*	1.996	2019	2	102.00
Treskes, R.; Wildbergh, T.; Schalij, M.; Scherptong, R.	Expectations and perceived barriers to widespread implementation of e-health in cardiology practice: Results from a national survey in the Netherlands	*Neth. Heart J.*	1.972	2019	2	102.00
Koole, M.A.C.; Kauw, D.; Winter, M.M.; Dohmen, D.A.J.; Tulevski, I.I.; de Haan, R.; Somsen, G.A.; Schijven, M.P.; Robbers-Visser, D.; Mulder, B.J.M.; Bouma, B.J.; Schuuring, M.J.	First real-world experience with mobile health telemonitoring in adult patients with congenital heart disease	*Neth. Heart J.*	1.972	2019	2	102.00
Kim, S.C.; Shaw, B.R.; Shah, D.V.; Hawkins, R.P.; Pingree, S.; McTavish, F.M.; Gustafson, D.H.	Interactivity, presence, and targeted patient care: Mapping e-health intervention effects over time for cancer patients with depression	*Health Communication*	1.846	2019	2	102.00
Hang, L.; Choi, E.; Kim, D.H.	A novel EMR integrity management based on a medical blockchain platform in hospital	*Electronics*	1.764	2019	2	102.00
Dao, J.; Spooner, C.; Lo, W.; Harris, M.	Factors influencing self-management in patients with type 2 diabetes in general practice: A qualitative study	*Australian Journal of Primary Health*	1.024	2019	2	102.00
Lee, H.; Sullivan, S.J.; Schneiders, A.G.; Ahmed, O.H.; Balasundaram, A.P.; Williams, D.; Meeuwisse, W.H.; McCrory, P.	Smartphone and tablet apps for concussion road warriors (team clinicians): A systematic review for practical users	*Br. J. Sports Med.*	0	2015	42	102.00
[95] Kebede, M.; Peters, M.; Heise, T.; Pischke, C.	Comparison of three meta-analytic methods using data from digital interventions on type 2 diabetes	*Diabetes, Metabolic Syndrome and Obesity*	0	2019	2	102.00
Kooistra, L.; Ruwaard, J.; Wiersma, J.; Van Oppen, P.; Van Der Vaart, R.; Van Gemert-Pijnen, J.; Riper, H.	Development and initial evaluation of blended cognitive behavioural treatment for major depression in routine specialized mental health care	*Internet Interv.*	0	2016	32	102.00
Petrakis, E.G.; Sotiriadis, S.; Soultanopoulos, T.; Renta, P.T.; Buyya, R.; Bessis, N.	Internet of Things as a Service (iTaaS): Challenges and solutions for management of sensor data on the cloud and the fog	*Internet of Things*	0	2018	12	102.00
Rajan, S.P.	Review and investigations on future research directions of mobile based telecare system for cardiac surveillance	*J. Appl. Res. Technol.*	0	2015	42	102.00
[65] Bricker, J.; Mull, K.; McClure, J.; Watson, N.; Heffner, J.	Improving quit rates of web-delivered interventions for smoking cessation: Full-scale randomized trial of WebQuit.org versus Smokefree.gov	*Addiction*	6.851	2018	11	101.01
Van Den Berg, M.; Crotty, M.; Liu, E.; Killington, M.; Kwakkel, G.; Van Wegen, E.	Early supported discharge by caregiver-mediated exercises and e-health support after stroke: A proof-of-concept trial	*Stroke*	6.046	2016	31	101.01
Al-Sharhan, S.; Omran, E.; Lari, K.	An integrated holistic model for an eHealth system: A national implementation approach and a new cloud-based security model	*International Journal of Information Management*	5.063	2019	1	101.01
[82] Liu, X., Zhou, Y.; Wang, Z.	Can the development of a patient’s condition be predicted through intelligent inquiry under the e-health business mode? Sequential feature map-based disease risk prediction upon features selected from cognitive diagnosis Big Data	*Int. J. Inf. Manag.*	5.063	2019	1	101.01
Danaher, B.; Tyler, M.; Crowley, R.; Brendryen, H.; Seeley, J.	Outcomes and device usage for fully automated internet interventions designed for a smartphone or personal computer: The MobileQuit smoking cessation randomized controlled trial	*J. Med. Internet Res.*	4.945	2019	1	101.00
Din, H.; Mcdaniels-Davidson, C.; Nodora, J.; Madanat, H.	Profiles of a health information-seeking population and the current digital divide: Cross-sectional analysis of the 2015–2016 California health interview survey	*J. Med. Internet Res.*	4.945	2019	1	101.00
Holter, M.; Johansen, A.; Ness, O.; Brinkmann, S.; Hoybye, M.; Brendryen, H.	Qualitative interview studies of working mechanisms in electronic health: tools to enhance study quality	*J. Med. Internet Res.*	4.945	2019	1	101.00
Kuipers, E.; Poot, C.C.; Wensing, M.; Chavannes, N.H.; de Smet, P.A.G.M.; Teichert, M.	Self-Management Maintenance Inhalation Therapy with eHealth (SELFIE): Observational study on the use of an electronic monitoring device in respiratory patient care and research	*J. Med. Internet Res.*	4.945	2019	1	101.00
Hsia, T.-L.; Chiang, A.-J.; Wu, J.-H.; Teng, N.N.; Rubin, A.D.	What drives e-health usage? Integrated institutional forces and top management perspectives	*Computers in Human Behavior*	4.306	2019	1	101.00
Klaib, A.F.; Nuser, M.S.	Evaluating EHR and health care in Jordan according to the international Health Metrics Network (HMN) framework and standards: A case study of Hakeem	*Access, IEEE*	4.098	2019	1	101.00
Menon, A.; Gray, L.; Fatehi, F.; Bird, D.; Darssan, D.; Karunanithi, M.; Russell, A.	Mobile-based insulin dose adjustment for type 2 diabetes in community and rural populations: Study protocol for a pilot randomized controlled trial	*Ther. Adv. Endocrinol. Metab.*	3.543	2019	1	101.00
Smith, B.; Magnani, J.	New technologies, new disparities: The intersection of electronic health and digital health literacy	*International Journal of Cardiology*	3.471	2019	1	101.00
Gibbs, J.; Gkatzidou, V.; Tickle, L.; Manning, S.; Tilakkumar, T.; Hone, K.; Ashcroft, R.; Sonnenberg, P.; Sadiq, S.; Estcourt, C.	“Can you recommend any good STI apps?” A review of content, accuracy and comprehensiveness of current mobile medical applications for STIs and related genital infections	*Sex Transm Infect*	3.365	2017	21	101.00
Heard, K.; Hughes, S.; Mughal, N.; Azadian, B.; Moore, L.	Evaluating the impact of the ICNET^®^ clinical decision support system for antimicrobial stewardship	*Antimicrobial Resistance and Infection Control*	3.224	2019	1	101.00
[91] Wild, T.; Fromberger, P.; Jordan, K.; Müller, I.; Müller, J.	Web-based health services in forensic psychiatry: A review of the use of the internet in the treatment of child sexual abusers and child sexual exploitation material offenders	*Front Psychiatry*	3.164	2019	1	101.00
Miramontes, R.; Aquino, R.; Flores, A.; Rodríguez, G.; Anguiano, R., Ríos, A.; Edwards, A.	PlaIMoS: A remote mobile healthcare platform to monitor cardiovascular and respiratory variables	*Sensors*	3.031	2017	21	101.00
Woods, S.; Sullivan, K.	Lower neurocognitive functioning disrupts the effective use of internet-based health resources in HIV disease: the mediating effects of general health literacy capacity	*AIDS Behav.*	2.908	2019	1	101.00
Young, C.; Campolonghi, S.; Ponsonby, S.; Dawson, S.L.; O’Neil, A.; Kay-Lambkin, F.; McNaughton, S.A.; Berk, M.; Jacka, F.N.	Supporting engagement, adherence, and behavior change in online dietary interventions	*Journal of Nutrition Education and Behavior*	2.869	2019	1	101.00
Walsh, K; Pryor, T.; Reynolds, K.; Walker, J.	Searching for answers: How well do depression websites answer the public’s questions about treatment choices?	*Patient Educ. Couns.*	2.821	2019	1	101.00
Cipolletta, S.; Mocellin, D.	Online counseling: An exploratory survey of Italian psychologists’ attitudes towards new ways of interaction	*Psychother. Res.*	2.788	2018	11	101.00
Olivero, E.; Bert, F.; Thomas, R.; Scarmozzino, A.; Raciti, I.; Gualano, M.; Siliquini, R.	E-tools for hospital management: An overview of smartphone applications for health professionals	*International Journal of Medical Informatics*	2.721	2019	1	101.00
Calvillo-Arbizu, J.; Roa-Romero, L.M.; Estudillo-Valderrama, M.A.; Salgueira-Lazo, M.; Aresté-Fosalba, N.; Del Castillo-Rodríguez, N.L.; González-Cabrera, F.; Marrero-Robayna, S.; De-La Manzana, V.L.; Román-Martínez, I.	User-centred design for developing e-health system for renal patients at home (AppNephro)	*International Journal of Medical Informatics*	2.721	2019	1	101.00
Haddad, S.; Souza, R.; Cecatti, J.	Mobile technology in health (mHealth) and antenatal care—searching for apps and available solutions: A systematic review	*International Journal of Medical Informatics*	2.721	2019	1	101.00
Miñarro-Giménez, J.; Cornet, R.; Jaulent, M.; Dewenter, H.; Thun, S., Gøeg, K.; Karlsson, D.; Schulz, S.	Quantitative analysis of manual annotation of clinical text samples	*International Journal of Medical Informatics*	2.721	2019	1	101.00
Lauridsen, S.; Braae, U.C.; Ngowi, H.A.; Johansen, M.V.	Impacts of using the electronic-health education program “The Vicious Worm” for prevention of Taenia solium	*Acta Tropica*	2.629	2019	1	101.00
Heger, I; Deckers, K.; Van Boxtel, M.; De Vugt, M.; Hajema, K., Verhey, F.; Köhler, S.	Dementia awareness and risk perception in middle-aged and older individuals: Baseline results of the MijnBreincoach survey on the association between lifestyle and brain health	*BMC Public Health*	2.567	2019	1	101.00
Kloek, C.; Van Tilburg, M.; Staal, J.; Veenhof, C.; Bossen, D.	Development and proof of concept of a blended physiotherapeutic intervention for patients with non-specific low back pain	*Physiotherapy (United Kingdom)*	2.534	2019	1	101.00
Novak, S.; Djordjevic, N.	Information system for evaluation of healthcare expenditure and health monitoring	*Physica A*	2.500	2019	1	101.00
[101] Amin, R.; Islam, S.K.H.; Biswas, G.P.; Khan, M.K.; Li, X.	Cryptanalysis and enhancement of anonymity preserving remote user mutual authentication and session key agreement scheme for e-health care systems	*J. Med. Syst.*	2.415	2015	41	101.00
[51] Li, X.; Niu, J.; Karuppiah, M.; Kumari, S.; Wu, F.	Secure and efficient two-factor user authentication scheme with user anonymity for network based e-health care applications	*J. Med. Syst.*	2.415	2016	31	101.00
Wiljer, D.; Charow, R.; Costin, H.; Sequeira, L.; Anderson, M., Strudwick, G.; Tripp, T.; Crawford, A.	Defining compassion in the digital health age: Protocol for a scoping review	*BMJ Open*	2.376	2019	1	101.00
Mangin, D.; Parascandalo, J., Khudoyarova, O., Agarwal, G., Bismah, V.; Orr, S.	Multimorbidity, eHealth and implications for equity: A cross-sectional survey of patient perspectives on eHealth	*BMJ Open*	2.376	2019	1	101.00
Krusche, A.; Bradbury, K.; Corbett, T.; Barnett, J.; Stuart, B.; Yao, G.L.; Bacon, R.; Böhning, D.; Cheetham-Blake, T.; Eccles, D.; Foster, C.; Geraghty, A.W.A.; Leydon, G.; Müller, A.; Neal, R.D.; Osborne, R.; Rathod, S.; Richardson, A.; Sharman, G.; Summers, K.; Watson, E.; Wilde, L.; Wilkinson, C.; Yardley, L.; Little, P.	Renewed: Protocol for a randomised controlled trial of a digital intervention to support quality of life in cancer survivors	*BMJ Open*	2.376	2019	1	101.00
Zayyad, M.A.; Toycan, M.	Factors affecting sustainable adoption of e-health technology in developing countries: An exploratory survey of Nigerian hospitals from the perspective of healthcare professionals	*PEERJ*	2.353	2018	11	101.00
Zanutto, A.	“Two clicks and I’m in!” Patients as co-actors in managing health data through a personal health record infrastructure	*Health Informatics J.*	2.297	2019	1	101.00
Mauco, K.L.; Scott, R.E.; Mars, M.	Critical analysis of e-health readiness assessment frameworks: Suitability for application in developing countries	*J. Telemed. Telecare*	2.229	2018	11	101.00
Olayiwola, J.N.; Potapov, A.; Gordon, A.; Jurado, J.; Magana, C.; Knox, M.; Tuot, D.	Electronic consultation impact from the primary care clinician perspective: Outcomes from a national sample	*J. Telemed. Telecare*	2.229	2019	1	101.00
[103] Kompara, M.; Kumari, S.; Hölbl, M.	Analysis and improvement of a secure key management protocol for e-health applications	*Computers & Electrical Engineering*	2.189	2019	1	101.00
Nymberg, V.M.; Bolmsjo, B.B.; Wolff, M.; Calling, S.; Gerward, S.; Sandberg, M.	“Having to learn this so late in our lives horizontal…” Swedish elderly patients’ beliefs, experiences, attitudes and expectations of e-health in primary health care	*Scand J Prim Health Care*	2.095	2019	1	101.00
Gupta, S.; Cheung, V.L.S.; Kastner, M.; Straus, S.; Kaplan, A.; Boulet, L.-P.; Sale, J.E.M.	Patient preferences for a touch screen tablet-based asthma questionnaire	*Journal of Asthma*	2.081	2019	1	101.00
James, N.; Power, E.; Hogden, A.; Vucic, S.	Patients’ perspectives of multidisciplinary home-based e-health service delivery for motor neurone disease	*Disability and rehabilitation*	2.054	2019	1	101.00
Dang, S.; Ruiz, D.; Klepac, L.; Morse, S.; Becker, P.; Levy, C.; Kinosian, B.; Edes, T.	Key characteristics for successful adoption and implementation of home telehealth technology in Veterans Affairs home-based primary care: An exploratory study	*Telemedicine and e-Health*	1.996	2019	1	101.00
Botrugno, C.	Towards an ethics for telehealth	*Nurs Ethics*	1.957	2019	1	101.00
Petrellis, N.; Birbas, M.; Gioulekas, F.	On the design of low-cost IoT sensor node for e-health environments	*Electronics*	1.764	2019	1	101.00
Shin, S.Y.	Current status and future direction of digital health in Korea	*Korean J. Physiol. Pharmacol*	1.654	2019	1	101.00
Van Dooren, M.M.; Siriaraya, P.; Visch, V.; Spijkerman, R.; Bijkerk, L.	Reflections on the design, implementation, and adoption of a gamified eHealth application in youth mental healthcare	*Entertainment Computing*	1.297	2019	1	101.00
Kinnunen, U.-M.; Heponiemi, T.; Rajalahti, E.; Ahonen, O.; Korhonen, T.; Hyppönen, H.	Factors related to health informatics competencies for nurses—results of a national electronic health record survey	*CIN—Computers Informatics Nursing*	1.029	2019	1	101.00
Del Río Carral, M.; Schweizer, A.; Papon, A.; Santiago-Delefosse, M.	Connected objects and health applications: Exploratory study on attitudes, use (or non-use) and contexts of use [Les objets connectés et applications de santé: étude exploratoire des perceptions, usages (ou non) et contextes d’usage]	*Pratiques Psychologiques*	0.196	2019	1	101.00
Kiberu, V.M.; Mars, M.; Scott, R.E.	Barriers and opportunities to implementation of sustainable e-health programmes in Uganda: A literature review	*AFR. J. Prim. Health Care Fam. Med.*	0	2017	21	101.00
Delgado, J.A.M.; Alonso, F.J.M.; Boquet, E.M.; de Tomás, J.F.Á.; Díez, J.M.C.	Competencias digitales clave de los profesionales sanitarios	*Educación Médica*	0	2019	1	101.00
Dai, J.; Granikov, V.; El Sherif, R.; Grguric, E.; Turcotte, E.; Pluye, P.	Patient Information Aid: An innovative educational program to improve outcomes of online consumer health information	*Education For Information*	0	2019	1	101.00
Kose, T.; Oymak, C.	E-health in Turkey: An analysis of consumer activities	*Health and Technology*	0	2019	1	101.00
[100] Ramu, G.; Reddy, B.E.; Jayanthi, A.; Prasad, L.V.N.	Fine-grained access control of EHRs in cloud using CP-ABE with user revocation	*Health and Technology*	0	2019	1	101.00
Merlo, C.; Akle, A.A.; Llaria, A.; Terrasson, G.; Villeneuve, E.; Pilnière, V.	Proposal of a user-centred approach for CPS design: Pillbox case study	*IFAC- PapersOnLine*	0	2019	1	101.00
Omotosho, A.; Ayegba, P.; Emuoyibofarhe, J.; Meinel, C.	Current state of ICT in healthcare delivery in developing countries	*International Journal of Online and Biomedical Engineering*	0	2019	1	101.00
Van Der Meulen, H.; Mccashin, D.; O’Reilly, G.; Coyle, D.	Using computer games to support mental health interventions: Naturalistic deployment study	*JMIR Mental Health*	0	2019	1	101.00
Moor, C.; Gür-Demirel, Y.; Wijsenbeek, M.	Feasibility of a comprehensive home monitoring program for sarcoidosis	*J. Pers. Med.*	0	2019	1	101.00
Maunder, K.; Walton, K.; Williams, P.; Ferguson, M.; Beck, E.	Strategic leadership will be essential for dietitian eHealth readiness: A qualitative study exploring dietitian perspectives of eHealth readiness	*Nutrition & Dietetics: The Journal of The Dietitians Association of Australia*	0	2019	1	101.00
Naarding, P.; Marijnissen, R.; Westerhof, G.	Digital psychiatry [Digitale psychiatrie]	*Tijdschr. Psychiatr.*	0	2019	1	101.00
Licari, A.; Ferrante, G.; Gian Luigi Marseglia, M.D.; Giovanni Corsello, M.D.; Grutta, S.L.	What is the impact of innovative electronic health interventions in improving treatment adherence in asthma? The pediatric perspective	*J. Allergy Clin. Immunol. Pract.*	7.550	2019	0	100.01
Garcia, S.; Wortman, K.; Cella, D.; Wagner, L.; Bass, M.; Kircher, S.; Pearman, T.; Penedo, F.	Implementing electronic health record–integrated screening of patient-reported symptoms and supportive care needs in a comprehensive cancer center	*Cancer*	6.164	2019	0	100.01
Carter, J.; Seed, P.T.; Watson, H.A.; David, A.L.; Sandall, J.; Shennan, A.H.; Tribe, R.M.	Development and validation of prediction models for the QUiPP App v.2: A tool for predicting preterm birth in women with symptoms of threatened preterm labor	*Ultrasound in obstetrics & gynecology*	5.595	2019	0	100.01
Delanty, N.; White, M.; Benson, K.; McCormack, M.; Heavin, S.; Comerford, E.; Gangadharan, N.; Power, K.; Dunleavey, B.; El-Naggar, H.; Doherty, C.; Greally, M.; Cavalleri, G.; Fitzsimons, M.	Development of a genomics module within an epilepsy-specific electronic health record: Toward genomic medicine in epilepsy care	*Epilepsia*	5.562	2019	0	100.01
Webber, E.; Brick, D.; Scibilia, J.; Dehnel, P.; Weinberg, S.; Alexander, G.; Beyer, E.; Hamling, A.; Kirkendall, E.; Lighter, D.; Mann, A.; Morgan, S.; Shelov, E.; Wright, J.; Alverson, D.; Chan, F.; Van Cain, M.; Krams, L.; Altman, R.; Bondi, S.; Fanaroff, J.; Narang, S.; Oken, R.; Rusher, J.; Santucci, K.; Scott, S.; Ake, J.; Alexander, J.; Bodnar, C.; Curfman, A., Herendeen, N.; Kahn, J.; McSwain, S.; Garber, K.; Calabrese, T., Council On Clinical Information Technology; Committee On Medical Liability And Risk Management; Section On Telehealth Care.	Electronic communication of the health record and information with pediatric patients and their guardians	*Pediatrics*	5.401	2019	0	100.01
Yi, C.; Cai, J.	Delay-dependent priority-aware transmission scheduling for e-health networks: A mechanism design approach	*IEEE Trans. Veh. Technol.*	5.339	2019	0	100.01
Fang, S.H.; Li, C.C.; Lu, W.C.; Xu, Z.; Chien, Y.R.	Enhanced device-free human detection: Efficient learning from phase and amplitude of channel state information	*IEEE Trans. Veh. Technol.*	5.339	2019	0	100.01
Elfstrom, K.M.; Sundstrom, K.; Andersson, S.; Bzhalava, Z.; Carlsten Thor, A.; Gzoul, Z.; Ohman, D.; Lamin, H.; Eklund, C.; Dillner, J.; Tornberg, S.	Increasing participation in cervical screening by targeting long-term nonattenders: Randomized health services study	*International Journal of Cancer*	4.982	2019	0	100.00
Cullinan, N.; Villani, A.; Mourad, S.; Somers, G.; Reichman, L.; Van Engelen, K.; Stephens, D.; Weksberg, R.; Foulkes, W.; Malkin, D.; Grant, R; Goudie, C.	An eHealth decision-support tool to prioritize referral practices for genetic evaluation of patients with Wilms tumor	*International Journal of Cancer*	4.982	2019	0	100.00
Den Bakker, C.M.; Huirne, J.A.; Schaafsma, F.G.; De Geus, C., Bonjer, H.J.; Anema, J.R.	Electronic health program to empower patients in returning to normal activities after colorectal surgical procedures: Mixed-methods process evaluation alongside a randomized controlled trial	*J. Med. Internet Res.*	4.945	2019	0	100.00
Paulsen, M.; Varsi, C.; Paur, I.; Tangvik, R.; Andersen, L.	Barriers and facilitators for implementing a decision support system to prevent and treat disease-related malnutrition in a hospital setting: Qualitative study	*J. Med. Internet Res.*	4.945	2019	0	100.00
Ariens, L.F.M.; Schussler-Raymakers, F.M.L.; Frima, C.; Flinterman, A.; Hamminga, E.; Arents, B.W.M.; Bruijnzeel-Koomen, C.A.F.M.; De Bruin-Weller, M.S.; Van Os-Medendorp, H.	Barriers and facilitators to eHealth use in daily practice: Perspectives of patients and professionals in dermatology	*J. Med. Internet Res.*	4.945	2017	20	100.00
Ijzerman, R.V.H.; Van Der Vaart, R.; Evers, A.W.M.	Internet-based cognitive behavioral therapy among psychologists in a medical setting: A survey on implementation	*J. Med. Internet Res.*	4.945	2019	0	100.00
Moore, G.; Wilding, H.; Gray, K.; Castle, D.	Participatory methods to engage health service users in the development of electronic health resources: Systematic review	*J Med. Internet Res.*	4.945	2019	0	100.00
Feijt, M.; De Kort, Y.; Bongers, I.; IJsselsteijn, W.	Perceived drivers and barriers to the adoption of eMental health by psychologists: The construction of the levels of adoption of eMental health model	*J. Med. Internet Res.*	4.945	2018	10	100.00
Sjöström, A.; Hörnsten, A.; Hajdarevic, S.; Emmoth, A.; Isaksson, U.	Primary health care nurses’ experiences of consultations with internet-informed patients: qualitative study	*J. Med. Internet Res.*	4.945	2019	0	100.00
Abbott-Garner, P.; Richardson, J.; Jones, R.	The impact of superfast broadband, tailored booklets for households, and discussions with general practitioners on personal electronic health readiness: Cluster factorial quasi-randomized control trial	*J Med. Internet Res.*	4.945	2019	0	100.00
Kip, H.; Kelders, S.M.; Bouman, Y.H.A.; Van Gemert-Pijnen, L.J.E.W.C.	The importance of systematically reporting and reflecting on eHealth development: Participatory development process of a virtual reality application for forensic mental health care	*J. Med. Internet Res.*	4.945	2019	0	100.00
Schofield, P.; Shaw, T.; Pascoe, M.	Toward comprehensive patient-centric care by integrating digital health technology with direct clinical contact in Australia	*J. Med. Internet Res.*	4.945	2019	0	100.00
Neijenhuijs, K.I.; Van Der Hout, A.; Veldhuijzen, E.; Scholten-Peeters, G.G.M.; Van Uden-Kraan, C.F.; Cuijpers, P.; Verdonck-De Leeuw, I.M.	Translation of the eHealth Impact Questionnaire for a population of Dutch electronic health users: Validation study	*J. Med. Internet Res.*	4.945	2019	0	100.00
Gray, C.; Gravesande, J.; Hans, P.; Nie, J.; Sharpe, S.; Loganathan, M.; Lyons, R.; Cott, C.	Using exploratory trials to identify relevant contexts and mechanisms in complex electronic health interventions: Evaluating the electronic patient-reported outcome tool	*J. Med. Internet Res.*	4.945	2019	0	100.00
Ospina-Pinillos, L.; Davenport, T.; Diaz, A.; Navarro-Mancilla, A.; Scott, E.; Hickie, I.	Using participatory design methodologies to co-design and culturally adapt the Spanish version of the mental health eClinic: Qualitative study	*J. Med. Internet Res.*	4.945	2019	0	100.00
Carroll, J.; Tobin, J.; Luque, A.; Farah, S.; Sanders, M.; Cassells, A.; Fine, S.; Cross, W.; Boyd, M.; Holder, T.; Thomas, M.; Overa, C.; Fiscella, K.	“Get Ready and Empowered About Treatment” (GREAT) Study: A pragmatic randomized controlled trial of activation in persons living with HIV	*J. Gen. Intern. Med.*	4.606	2019	0	100.00
Mapar, M.; Jafari, M.; Mansouri, N.; Arjmandi, R.; Azizinejad, R.; Ramos, T.	Sustainability indicators for municipalities of megacities: Integrating health, safety and environmental performance	*Ecological Indicators*	4.49	2017	20	100.00
Maillart, E.; Labauge, P.; Cohen, M.; Maarouf, A.; Vukusic, S.; Donze, C.; Gallien, P.; De Seze, J.; Bourre, B.; Moreau, T.; Louapre, C.; Mayran, P.; Bieuvelet, S.; Vallee, M.; Bertillot, F.; Klaeyle, L.; Argoud, A.-L.; Zinai, S.; Tourbah, A.	MSCopilot, a new multiple sclerosis self-assessment digital solution: Results of a comparative study versus standard tests	*Eur. J. Neurol.*	4.387	2019	0	100.00
Li, D.; Brown, C.; Gallo, C.; Morgan, E.; Sullivan, P.; Young, S.; Mustanski, B.	Design considerations for implementing eHealth behavioral interventions for HIV prevention in evolving sociotechnical landscapes	*Curr. HIV/AIDS Rep.*	4.382	2019	0	100.00
[87] Jacob, C.; Sanchez-Vazquez, A.; Ivory, C.	Clinicians’ role in the adoption of an oncology decision support app in Europe and its implications for organizational practices: Qualitative case study	*JMIR mHealth and uHealth*	4.301	2019	0	100.00
Knitza, J.; Tascilar, K.; Messner, E.-M.; Meyer, M.; Vossen, D.; Pulla, A.; Bosch, P.; Kittler, J.; Kleyer, A.; Sewerin, P.; Mucke, J.; Haase, I., Simon, D.; Krusche, M.	German mobile apps in rheumatology: Review and analysis using the Mobile Application Rating Scale (MARS)	*JMIR mHealth and uHealth*	4.301	2019	0	100.00
Mountford, N.	Managing by proxy: Organizational networks as institutional levers in evolving public good markets	*J. Bus. Res.*	4.028	2019	0	100.00
[86] Yang, H.; Guo, X.; Wu, T.	Exploring the influence of the online physician service delivery process on patient satisfaction	*Decision Support Systems*	3.847	2015	40	100.00
Farid, S.	Conceptual framework of the impact of health technology on healthcare system	*Front Pharmacol*	3.845	2019	0	100.00
Niemelä, R.; Pikkarainen, M.; Ervasti, M.; Reponen, J.	The change of pediatric surgery practice due to the emergence of connected health technologies	*Technol. Forecast. Soc. Change*	3.815	2019	0	100.00
Canonica, G.; Bachert, C.; Hellings, P.; Ryan, D.; Valovirta, E.; Wickman, M.; De Beaumont, O.; Bousquet, J.	Allergen Immunotherapy (AIT): A prototype of Precision Medicine	*World Allergy Organization Journal*	3.684	2015	40	100.00
Peeters, J.M.; Krijgsman, J.W.; Brabers, A.E.; De Jong, J.D.; Friele, R.D.	Use and uptake of eHealth in general practice: A cross-sectional survey and focus group study among health care users and general practitioners	*JMIR Medical Informatics*	3.188	2016	30	100.00
[93] Van Der Linden, S.; Sitskoorn, M.; Rutten, G.J.; Gehring, K.	Feasibility of the evidence-based cognitive telerehabilitation program Remind for patients with primary brain tumors	*J. Neurooncol.*	3.129	2018	10	100.00
Bervell, B.; Al-Samarraie, H.	A comparative review of mobile health and electronic health utilization in sub-Saharan African countries	*Soc. Sci. Med.*	3.087	2019	0	100.00
[98] Boussada, R.; Hamdane, B.; Elhdhili, M.E.; Saidane, L.A.	Privacy-preserving aware data transmission for IoT-based e-health	*Computer Networks*	3.03	2019	0	100.00
Mondal, S.; Mukherjee, N.	An efficient reachability query based pruning algorithm in e-health scenario	*J. Biomed. Inf.*	2.95	2019	0	100.00
Perazzo, J.; Reyes, D.; Webel, A.	A systematic review of health literacy interventions for people living with HIV	*AIDS Behav.*	2.908	2017	20	100.00
Carmel, A.; Cornelius-Schecter, A.; Frankel, B.; Jannat-Khah, D.; Sinha, S.; Pelzman, F.; Safford, M.	Evaluation of the Patient Activated Learning System (PALS) to improve knowledge acquisition, retention, and medication decision making among hypertensive adults: Results of a pilot randomized controlled trial	*Patient Educ. Couns.*	2.821	2019	0	100.00
Chang, Y.W.; Hsu, P.Y.; Wang, Y.; Chang, P.Y.	Integration of online and offline health services: The role of doctor-patient online interaction	*Patient Educ. Couns.*	2.821	2019	0	100.00
Fang, S.Y.; Wang, Y.L.; Lu, W.H.; Lee, K.T.; Kuo, Y.L.; Fetzer, S.J.	Long-term effectiveness of an E-based survivorship care plan for breast cancer survivors: A quasi-experimental study	*Patient Educ. Couns.*	2.821	2019	0	100.00
Helle, C.; Hillesund, E.; Wills, A.; Øverby, N.	Examining the effects of an eHealth intervention from infant age 6 to 12 months on child eating behaviors and maternal feeding practices one year after cessation: The Norwegian randomized controlled trial Early Food for Future Health	*Plos One*	2.776	2019	0	100.00
Denis, F.; Voog, E.; Pointreau, Y.; Bourgeois, H.; Seegers, V.; Le Du, K.	Prospective study of a web-mediated management of febrile neutropenia related to chemotherapy (Bioconnect)	*Support Care Cancer*	2.754	2019	0	100.00
Sadegh, S.S.; Saadat, P.K.; Sepehri, M.M.; Assadi, V.	A framework for m-health service development and success evaluation	*International Journal of Medical Informatics*	2.721	2018	10	100.00
Frontoni, E.; Mancini, A.; Baldi, M.; Paolanti, M.; Moccia, S.; Zingaretti, P.; Landro, V.;Misericordia, P.	Sharing health data among general practitioners: The Nu.Sa. project	*International Journal of Medical Informatics*	2.721	2019	0	100.00
Gu, D.; Li, T.; Wang, X.; Yang, X.; Yu, Z.	Visualizing the intellectual structure and evolution of electronic health and telemedicine research	*International Journal of Medical Informatics*	2.721	2019	0	100.00
Kagawa, R.; Shinohara, E.; Imai, T.; Kawazoe, Y.; Ohe, K.	Bias of inaccurate disease mentions in electronic health record-based phenotyping	*International Journal of Medical Informatics*	2.721	2019	0	100.00
Parks, R.; Wigand, R.; Othmani, M.; Serhier, Z.; Bouhaddou, O.	Electronic health records implementation in Morocco: Challenges of silo efforts and recommendations for improvements	*International Journal of Medical Informatics*	2.721	2019	0	100.00
Krijnen-De Bruin, E.; Muntingh, A.; Hoogendoorn, A.; Van Straten, A.; Batelaan, N.; Maarsingh, O.; Van Balkom, A.; Van Meijel, B.	The GET READY relapse prevention programme for anxiety and depression: A mixed-methods study protocol	*BMC Psychiatry*	2.666	2019	0	100.00
Habboushe, J.; Altman, C.; Lip, G.	Time trends in use of the CHADS 2 and CHA 2 DS 2 VASc scores, and the geographical and specialty uptake of these scores from a popular online clinical decision tool and medical reference	*International Journal of Clinical Practice*	2.613	2019	0	100.00
Droes, R.M.; Van Rijn, A.;Rus, E.; Dacier, S.; Meiland, F.	Utilization, effect, and benefit of the individualized Meeting Centers Support Program for people with dementia and caregivers	*Clin. Interv. Aging.*	2.585	2019	0	100.00
Berger, M.; Steinberg, D.; Askew, S.; Gallis, J.; Treadway, C.; Egger, J.; Kay, M.; Batch, B.; Finkelstein, E.; Devries, A.; Brewer, A.; Bennett, G.	The Balance protocol: A pragmatic weight gain prevention randomized controlled trial for medically vulnerable patients within primary care	*BMC Public Health*	2.567	2019	0	100.00
Ohana, S.; Barnoy, S.	Israeli e-patients’ Informational Needs	*Nurs. Outlook*	2.54	2019	0	100.00
Albarrak, A.I.; Mohammed, R.; Almarshoud, N.; Almujalli, L.; Aljaeed, R.; Altuwaijiri, S.; Albohairy, T.	Assessment of physician’s knowledge, perception and willingness of telemedicine in Riyadh region, Saudi Arabia	*J. Infect. Public Health*	2.487	2019	0	100.00
Zhou, J.; Fan, T.	Understanding the factors influencing patient e-health literacy in online health communities (OHCs): A social cognitive theory perspective	*Int. J. Environ. Res Public Health*	2.468	2019	0	100.00
Rathnayake, S.; Senevirathna, A.	Self-reported eHealth literacy skills among nursing students in Sri Lanka: A cross-sectional study	*Nurse Educ Today*	2.442	2019	0	100.00
[102] Waschkau, A.; Wilfling, D.; Steinhaeuser, J.	Are Big Data analytics helpful in caring for multimorbid patients in general practice?—a scoping review	*BMC Fam. Pract.*	2.431	2019	0	100.00
Firet, L.; De Bree, C.; Verhoeks, C.; Teunissen, D.; Lagro-Janssen, A.	Mixed feelings: General practitioners’ attitudes towards eHealth for stress urinary incontinence—a qualitative study	*BMC Fam. Pract.*	2.431	2019	0	100.00
Gagnon, M.-P.;Ndiaye, M.A.; Larouche, A.; Chabot, G.; Chabot, C., Buyl, R.; Fortin, J.-P.; Giguere, A.; Leblanc, A.; Legare, F.; Motulsky, A.;Sicotte, C.; Witteman, H.O.; Kavanagh, E.; Lepinay, F.; Roberge, J.; Deletroz, C.; Abbasgholizadeh-Rahimi, S.	Optimising patient active role with a user-centred eHealth platform (CONCERTO plus) in chronic diseases management: A study protocol for a pilot cluster randomised controlled trial	*BMJ Open*	2.376	2019	0	100.00
Beishuizen, C.; Akenine, U.; Barbera, M.; Rosenberg, A.; Fallah Pour, M.; Richard, E.; Soininen, H.; Mangialasche, F.; Kivipelto, M.; Pols, A.; Moll Van Charante, E.	Integrating nurses’ experiences with supporting behaviour change for cardiovascular prevention into a self-management internet platform in Finland and the Netherlands: A qualitative study	*BMJ Open*	2.376	2019	0	100.00
Kocher, A.; Simon, M.; Dwyer, A.A.; Villiger, P.M.; Kunzler-Heule, P.; De Geest, S.; Berben, L.; Nicca, D.	Developing a rare disease chronic care model: Management of systemic sclerosis (MANOSS) study protocol	*Journal of Advanced Nursing*	2.376	2019	0	100.00
Ladan, M.A.; Wharrad, H.; Windle, R.	eHealth adoption and use among healthcare professionals in a tertiary hospital in Sub-Saharan Africa: A Qmethodology study	*PEERJ*	2.353	2019	0	100.00
Huang, R.-C.; Silva, D.; Beilin, L.; Neppe, C.; Mackie, K.; Roffey, E.; Gibson, L.; D’vaz, N.;Christian, H.;Reid, C.; Prescott, S.	Feasibility of conducting an early pregnancy diet and lifestyle e-health intervention: The Pregnancy Lifestyle Activity Nutrition (PLAN) project	*J. Dev. Orig. Health Dis.*	2.34	2019	0	100.00
Vamos, C.A.; Griner, S.B.; Kirchharr, C.; Green, S.M.; Debate, R.; Daley, E.M.; Quinonez, R.B.; Boggess, K.A; Jacobs, T.; Christiansen, S.	The development of a theory-based eHealth app prototype to promote oral health during prenatal care visits	*Transl. Behav. Med.*	2.237	2019	0	100.00
[59] Devan, H.; Godfrey, H.K.; Perry, M.A.; Hempel, D.; Saipe, B.; Hale, L.; Grainger, R.	Current practices of health care providers in recommending online resources for chronic pain self-management	*J. Pain Res.*	2.236	2019	0	100.00
Vehko, T.; Hyppönen, H.; Puttonen, S.; Kujala, S.; Ketola, E.; Tuukkanen, J.; Aalto, A.-M.; Heponiemi, T.	Experienced time pressure and stress: electronic health records usability and information technology competence play a role	*BMC Med. Inform. Decis. Mak.*	2.067	2019	0	100.00
[92] Duruturk, N.; Özköslü, M.A.	Effect of tele-rehabilitation on glucose control, exercise capacity, physical fitness, muscle strength and psychosocial status in patients with type 2 diabetes: A double blind randomized controlled trial	*Prim. Care Diabetes*	2.008	2019	0	100.00
Van Schaik, P.; Thornhill, E.; Davies, M.; Flynn, D.; Kusev, P.	The use of information in online healthcare provider choice	*Int. J. Hum. Comput. Stud.*	2.006	2019	0	100.00
Arduini, D.; Zanfei, A.	An overview of scholarly research on public e-services? A meta-analysis of the literature	*Telecomm Policy*	2.000	2014	50	100.00
Doarn, C.; Zacharias, S.; Keck, C.; Tabangin, M.; Dealarcon, A.; Kelchner, L.	Design and implementation of an interactive website for pediatric voice therapy—the concept of in-between care: A telehealth model	*Telemedicine and e-Health*	1.996	2019	0	100.00
Doarn, C.R.; Dorogi, A.; Tikhtman, R.; Pallerla, H.; Vonder Meulen, M.B.	Opinions on the role of telehealth in a large Midwest academic health center: A case study	*Telemedicine and e-Health*	1.996	2019	0	100.00
Rebchuk, A.; Deptuck, H.; O’Neill, Z.; Fawcett, D.; Silverberg, N.; Field, T.	Validation of a novel telehealth administration protocol for the NIH toolbox-cognition battery	*Telemedicine and e-Health*	1.996	2019	0	100.00
Murchie, P.; Masthoff, J.; Walter, F.; Rahman, K.; Allan, J.; Burrows, N.; Proby, C.; Lee, A.; Johnston, M.; Durrani, A.; Depasquale, I.; Brant, B.; Neilson, A.; Meredith, F.; Treweek, S.; Hall, S.; McDonald, A.	Achieving Self-Directed Integrated Cancer Aftercare (ASICA) in melanoma: Protocol for a randomised patient-focused pilot trial of delivering the ASICA intervention as a means to earlier detection of recurrent and second primary melanoma	*Trials*	1.975	2019	0	100.00
Gerrits, O.	The future of healthcare has arrived: Who dares take up the challenge?	*Neth. Heart J.*	1.972	2019	0	100.00
Van Den Bosch, S.; Van De Voort, N.; Xi, T.; Kool, R.; Bergé, S.; Faber, M.	Oral and maxillofacial surgery is ready for patient-centred eHealth interventions—the outcomes of a scoping review	*International Journal of Oral and Maxillofacial Surgery*	1.961	2019	0	100.00
Warth, L.L.; Dyb, K.	eHealth initiatives; the relationship between project work and institutional practice	*BMC Health Serv, Res,*	1.932	2019	0	100.00
Heisler, M.; Choi, H.; Mase, R.; Long, J.A.; Reeves, P.J.	Effectiveness of technologically enhanced peer support in improving glycemic management among predominantly African American, low-income adults with diabetes	*Diabetes Educator*	1.910	2019	0	100.00
Ferguson, M.; Leighton, P.; Brandreth, M.; Wharrad, H.	Development of a multimedia educational programme for first-time hearing aid users: A participatory design	*International Journal of Audiology*	1.821	2018	10	100.00
Leung, C.; Shaipanich, T.	Current practice in the management of pulmonary nodules detected on computed tomography chest scans	*Can. Respir. J.*	1.803	2019	0	100.00
Shin, E.; Shim, J.-M.	Listen to doctors, friends, or both? Embedded they produce thick knowledge and promote health	*J. Health Commun.*	1.773	2019	0	100.00
[66] Jansson, M.M.; Harjumaa, M.; Puhto, A.-P.; Pikkarainen, M.	Healthcare professionals’ proposed eHealth needs in elective primary fast-track hip and knee arthroplasty journey: A qualitative interview study	*J. Clin. Nurs.*	1.757	2019	0	100.00
Kiberu, V.; Mars, M.; Scott, R.	Development of an evidence-based e-health readiness assessment framework for Uganda	*Health Information Management Journal*	1.742	2019	0	100.00
Aromatario, O.; Hoye, A.V.; Vuillemin, A.; Foucaut, A.-M.; Crozet, C.; Pommier, J.; Cambon, L.	How do mobile health applications support behaviour changes? A scoping review of mobile health applications relating to physical activity and eating behaviours	*Public Health*	1.696	2019	0	100.00
Lie, S., Karlsen, B., Graue, M. and Oftedal, B.	The influence of an eHealth intervention for adults with type 2 diabetes on the patient–nurse relationship: A qualitative study	*Scand. J. Caring Sci.*	1.642	2019	0	100.00
Momenipour, A.; Pennathur, P.	Balancing documentation and direct patient care activities: A study of a mature electronic health record system	*Int. J. Ind. Ergon.*	1.571	2019	0	100.00
[60] Zitkus, V.; Butkiene, R.; Butleris, R.; Maskeliunas, R.; Damasevicius, R.; Wozniak, M.	Minimalistic approach to coreference resolution in Lithuanian medical records	*Comput. Math. Methods Med.*	1.563	2019	0	100.00
Wingo, B.C.; Yang, D.; Davis, D.; Padalabalanarayanan, S.; Hopson, B.; Thirumalai, M.; Rimmer, J.H.	Lessons learned from a blended telephone/e-health platform for caregivers in promoting physical activity and nutrition in children with a mobility disability	*Disabil. Health J.*	1.471	2019	0	100.00
[46] Vyas, K.S.; Hambrick, H.R.; Shakir, A.; Morrison, S.D.; Tran, D.C.; Pearson, K.; Vasconez, H.C.; Mardini, S.; Gosman, A.A.; Dobke, M.; Granick, M.S.	A systematic review of the use of telemedicine in plastic and reconstructive surgery and dermatology	*Annals of Plastic Sugery*	1.448	2017	20	100.00
Brignone, L.; Edleson, J.	The dating and domestic violence app Rubric: Synthesizing clinical best practices and digital health app standards for relationship violence prevention smartphone apps	*Int. J. Hum. Comput. Interact.*	1.354	2019	0	100.00
[88] Triplett, K.; El-Behadli, A.; Masood, S.; Sullivan, S.; Desai, D.	Digital medicine program with pediatric solid organ transplant patients: Perceived benefits and challenges	*Pediatr. Transplant.*	1.326	2019	0	100.00
Garai, A.; Pentek, I.; Adamko, A.	Revolutionizing healthcare with IoT and cognitive, cloud-based telemedicine	*Acta Polytechnica Hungarica*	1.286	2019	0	100.00
Eden, R.; Burton-Jones, A.; Grant, J.; Collins, R.; Staib, A; Sullivan, C.	Digitising an Australian university hospital: Qualitative analysis of staff-reported impacts	*Australian Health Review*	1.228	2019	0	100.00
Ross, P.; Cross, R.	Rise of the e-nurse: The power of social media in nursing	*Contemp. Nurse*	1.216	2019	0	100.00
Castaneda, P.; Sales, A., Osborne, N. and Corriere, M.	Scope, themes, and medical accuracy of eHealth peripheral artery disease community forums	*Annals of Vascular Surgery*	1.179	2019	0	100.00
Charnock, V.	Electronic healthcare records and data quality	*Health Info. Libr. J.*	1.179	2019	0	100.00
Peterson, S.; Kuntz, C.; Roush, J.	Use of a modified treatment-based classification system for subgrouping patients with low back pain: Agreement between telerehabilitation and face-to-face assessments	*Physiother. Theory Pract.*	1.158	2019	0	100.00
Veldhuijzen, G.; Van Esch, A.; Klemt-Kropp, M.; Terhaar Sive Droste, J.; Drenth, J.	E-patient counseling trial (E-PACO): Computer based education versus nurse counseling for patients to prepare for colonoscopy	*JOVE—Journal of Visualized Experiments*	1.108	2019	0	100.00
Kerdjidj, O.; Amira, A.; Ghanem, K.; Ramzan, N.; Katsigiannis, S.; Chouireb, F.	An FPGA implementation of the matching pursuit algorithm for a compressed sensing enabled e-health monitoring platform	*Microprocessors and Microsystems*	1.045	2019	0	100.00
Serafica, R.; Inouye, J.; Lukkahatai, N.; Braginsky, N.; Pacheco, M.; Daub, K.	The use of mobile health to assist self-management and access to services in a rural community	*CIN—Computers Informatics Nursing*	1.029	2019	0	100.00
Abaza, H.; Marschollek, M.	mHealth application areas and technology combinations*. A comparison of literature from high and low/middle income countries	*Methods Inf. Med.*	1.024	2017	20	100.00
Souza, T.; Lobão, W.; Santos, C.; Almeida, M.; Moreira Júnior, E.	Factors associated with the acceptance of the influenza vaccine among health workers: Knowledge, attitude and practice [Fatores associados à aceitação da vacina influenza entre trabalhadores de saúde: conhecimento, atitude e prática]	*Cien. Saude. Colet.*	1.008	2019	0	100.00
Brown, L.J.; Jones, G.M.; Bond, M.J.	E-health: Psychosocial challenges for South Australian rural mental health consumers	*Rural Remote Health*	0.985	2019	0	100.00
Kips, J.; Lambert, J.; Ongenae, K.; De Sutter, A.; Verhaeghe, E.	Teledermatology in Belgium: A pilot study	*Acta Clinica Belgica*	0.960	2019	0	100.00
Clanchy, K.; Tweedy, S.; Trost, S.	The Adapted Physical Activity Program: A theory-driven, evidence-based physical activity intervention for people with brain impairment	*Brain Impairment*	0.958	2019	0	100.00
Berndt, R.-D.; Preik, P.; Takenga, C.	TeleDermatology: the teledermatological solution mSkin^®^ for daily practice	*Hautarzt*	0.828	2019	0	100.00
Jedamzik, S.	Digital health and nursing: the future is now [Digitale Gesundheit und Pflege: Die Zukunft ist jetzt]	*Unfallchirurg*	0.716	2019	0	100.00
Khan, I.; Xitong, G.; Ahmad, Z.; Shahzad, F.	Investigating factors impelling the adoption of e-health: A perspective of African expats in China	*Sage Open*	0.675	2019	0	100.00
Alghazo, J.M.	Intelligent security and privacy of electronic health records using biometric images	*Current Medical Imaging Reviews*	0.533	2019	0	100.00
De Leon-Castaneda, C.	Electronic health (e-health): A conceptual framework for implementation in health services	*Gac. Med. Mex.*	0.283	2019	0	100.00
Chadi, N.; Weisbaum, E.; Malboeuf-Hurtubise, C.; Kohut, S.A.; Viner, C.; Palaniyar, N.; Kaufman, M.; Locke, J.; Vo, D.X.	In-person vs. eHealth mindfulness-based intervention for adolescents with chronic illnesses: A pilot randomized trial	*Adolescent Psychiatry*	0	2019	0	100.00
Voutsidou, S.; Moraitis, E.; Jelastopoulou, E.; Sissouras, A.; Charalampous, G.	Electronic health applications in primary medical health care: Advantages and expectations	*Archives of Hellenic Medicine*	0	2019	0	100.00
Spyridaki, A.; Antonakos, I.; Apostolakis, I.; Tountas, I.	Investigation of the effectiveness of mobile health applications for chronic diseases	*Archives of Hellenic Medicine*	0	2019	0	100.00
Ramos, A.C.; Bouzas-Lorenzo, R.; del Olmo, A.M.; Buceta, B.B.	Opinión de los facultativos y usuarios sobre avances de la e-salud en atención primaria	*Aten. Prim.*	0	2019	0	100.00
Guarda, P.	“OK Google, am I sick?’’: Artificial intelligence, e-health, and data protection regulation	*Biolaw Journal-Rivista Di Biodiritto*	0	2019	0	100.00
Klocek, A.; Smahelova, M.; Knapova, L.; Elavsky, S.	GPs’ perspectives on eHealth use in the Czech Republic: A cross-sectional mixed-design survey study	*BJGP Open*	0	2019	0	100.00
Reddeman, L.; Bourgeois, N.; Angl, E.N.; Heinrich, M.; Hillier, L.; Finn, H.; Bosiak, B.; Agarwal, P.; Mawson, R.; Propp, R.; Ivers, N.M.	How should family physicians provide physical activity advice? Qualitative study to inform the design of an e-health intervention	*Can. Fam. Physician*	0	2019	0	100.00
Borries, T.M.; Dunbar, A.; Bhukhen, A.; Rismany, J.; Kilham, J., Feinn, R.; Meehan Sr., T.P.	The impact of telemedicine on patient self-management processes and clinical outcomes for patients with types I or II diabetes mellitus in the United States: A scoping review	*Diabetes & Metabolic Syndrome-Clinical Research & Reviews*	0	2019	0	100.00
Howarth, A.; Quesada, J.; Donnelly, T.; Mills, P.R.	The development of “Make One Small Change”: An e-health intervention for the workplace developed using the Person-Based Approach	*Digital Health*	0	2019	0	100.00
Krivenko V, N.; Elishev, V.G.; Kriventsova, L.A.	The impact of innovation on the performance of health care in the economic security system of the region	*Ekonomika Regiona-Economy of Region*	0	2019	0	100.00
Seckin, G.; Hughes, S.; Yeatts, D.; Degreve, T.	Digital pathways to positive health perceptions: Does age moderate the relationship between medical satisfaction and positive health perceptions among middle-aged and older internet users?	*Innov. Aging*	0	2019	0	100.00
Al-Sharekh, S.I.; Al-Shqeerat, K.H.A.	Security challenges and limitations in IoT environments	*IJCSNS*	0	2019	0	100.00
Azcarraga, J.; Raduban, J.; Christine Gendrano, M.; Azcarraga, A.	Identity concealment when uploading pictures of patients in a tele-medicine system	*International Journal of E-Health and Medical Communications*	0	2019	0	100.00
Khennou, F.; Chaoui, N.E.H.; Khamlichi, Y.I.	A migration methodology from legacy to new electronic health record based OpenEHR	*International Journal of E-Health and Medical Communications*	0	2019	0	100.00
Christie, H.L.; Martin, J.L.; Connor, J.; Tange, H.J.; Verhey, F.R., de; Vugt, M.E.; Orrell, M.	eHealth interventions to support caregivers of people with dementia may be proven effective, but are they implementation-ready?	*Internet Interv.*	0	2019	0	100.00
De Cicco, L.; Mascolo, S.; Palmisano, V.; Ribezzo, G.	Reducing the network bandwidth requirements for 360 degrees immersive video streaming	*Internet Technology Letters*	0	2019	0	100.00
[78] Schimmer, R.; Orre, C.; Oberg, U.; Danielsson, K.; Hornsten, A.	Digital person-centered self-management support for people with type 2 diabetes: Qualitative study exploring design challenges	*JMIR Diabetes*	0	2019	0	100.00
Goodall, G.; Ciobanu, I.; Taraldsen, K.; Sorgaard, J.; Marin, A., Draghici, R.; Zamfir, M.-V.; Berteanu, M.; Maetzler, W.; Serrano, J.A.	The use of virtual and immersive technology in creating personalized multisensory spaces for people living with dementia (SENSE-GARDEN): Protocol for a multisite before-after trial	*JMIR Res. Protoc.*	0	2019	0	100.00
Prasad, M.; Manjunath, C.; Murthy, A.K.; Sampath, A.; Jaiswal, S.; Mohapatra, A.	Integration of oral health into primary health care: A systematic review	*J. Family Med. Prim. Care*	0	2019	0	100.00
Bhatia, R.; Taneja, U.	Factors affecting indian consumers’ intention to use eHealth services	*Journal of Health Management*	0	2019	0	100.00
Farzana, S.; Islam, S.	Symmetric key-based patient controlled secured electronic health record management protocol	*Journal of High Speed Networks*	0	2019	0	100.00
Øvrelid, E; Halvorsen, M.	Supporting process innovation with lightweight IT at an emergency unit	*Journal of Integrated Design and Process Science*	0	2019	0	100.00
Eke, E.; Kisi, M.; Ugurluoglu, D.	A research on awareness of e-health practices	*Journal of Mehmet Akif Ersoy University Economics and Administrative Sciences Faculty*	0	2019	0	100.00
Anya, O.; Tawfik, H.; Alani, M.; Hu, J.	Cybersecurity design considerations for cross-boundary clinical decision support	*J Reliab. Intell. Environ.*	0	2019	0	100.00
Kruczek, A.	e-health—modern technologies in mental health care	*Postepy. Psychiatrii. I. Neurologii.*	0	2019	0	100.00
Costa, W.L.G.; Pereira, E.; Kontonatsios, G.	Use of smartphones for hypoglycaemia prediction—A feasibility study	*Procedia Comput Sci*	0	2019	0	100.00
Senges, E.; Guiot, D.; Chandon, J.-L.	Desired ageing well: Predictive validity for consumers aged 50–80	*Recherche Et Applications En Marketing-English Edition*	0	2019	0	100.00
Webers, C.; Beckers, E.; Boonen, A.; Van Eijk-Hustings, Y.; Vonkeman, H.; Van De Laar, M.; Van Tubergen, A.	Development, usability and acceptability of an integrated eHealth system for spondyloarthritis in the Netherlands (SpA-Net)	*RMD Open*	0	2019	0	100.00
Vesnic-Alujevic, L.; Pereira, A.G.; Breitegger, M.	Wearable sensors exploring EU policy narratives by engaging the extended peer community	*Tecnoscienza*	0	2019	0	100.00
Metz, M.J.; Veerbeek, M.A.; Elfeddali, I.; De Beurs, E.; Van Der Feltz-Cornelis, C.M.; Beekman, A.T.F.	Shared decision making in mental health care; evaluation of the added value for patients and clinicians	*Tijdschr. Psychiatr.*	0	2019	0	100.00
Allner, R.; Wilfling, D.; Kidholm, K.; Steinhäuser, J.	Telemedizinprojekte im ländlichen Raum Deutschlands. Eine systematische Bewertung mit dem “Modell zur Evaluation von telemedizinischen Anwendungen”. TT—[Telemedicine projects in rural areas of Germany. A systematic evaluation with the “M”	*Z. Evid. Fortbild. Qual. Gesundhwes*	0	2019	0	100.00
Scherer, M.; Szecsenyi, J.; Gerlach, F.	Digitalisation in medicine—Who proceeds and who looks behind a plea for a DEGAM digital strategy [Digitalisierung in der medizin—Wer schreitet voran, wer schaut hinterher? Ein plädoyer für eine DEGAM-digitalstrategie]	*Z. Allgemeinmed.*	0	2019	0	100.00
Gebre-Mariam, M.; Bygstad, B.	What can enterprise architecture do for healthcare? A framework of antecedents and benefits	*Electronic Government*	0	2019	0	100.00
Ivanova, O.; Wambua, S.; Mwaisaka, J.; Bossier, T.; Thiongo, M.; Michielsen, K.; Gichangi, P.	Evaluation of the ELIMIKA Pilot Project: Improving ART adherence among HIV positive youth using an eHealth intervention in Mombasa, Kenya	*African Journal of Reproductive Health*	0.547	2019	0	100.00
